# Baseline and Dynamic Clinical–Laboratory Risk Models for Early Progression or Death During PD-(L)1 Blockade in Advanced NSCLC: Development and Internal Validation

**DOI:** 10.3390/curroncol33100593

**Published:** 2026-09-30

**Authors:** Mehmet Fatih Ozbay, Mehmet Hunur, Sertac Vurgun, Suleyman Alkan, Sema Sezgin Goksu, Mustafa Karaca

**Affiliations:** Department of Medical Oncology, Akdeniz University Faculty of Medicine, 07058 Antalya, Türkiye

**Keywords:** non-small-cell lung cancer, immune checkpoint inhibitor, early progression, early death, EPS-4, neutrophil-to-lymphocyte ratio, lactate dehydrogenase, landmark analysis, prognostic model

## Abstract

Background: We developed clinical–laboratory models for early progression or death within 90 days (EPD90) during PD-1/PD-L1 blockade in advanced non-small cell lung cancer. EPD90 is a pragmatic early-risk endpoint, not a diagnosis of hyperprogressive disease. Methods: This retrospective single-centre cohort included patients treated between January 2018 and March 2026. The four-item baseline score, EPS-4, combined bone metastasis, lactate dehydrogenase (LDH), albumin and C-reactive protein. Among patients alive and progression-free at day 42, D1 added six-week changes in neutrophil-to-lymphocyte ratio (NLR), and D2 additionally included LDH changes. Internal validation used bootstrap resampling and repeated cross-validation. Results: Among 282 patients, 83 (29.4%) had EPD90. In the common baseline complete-case set (*n* = 252; 76 events), EPS-4 had an apparent area under the curve (AUC) of 0.729 (95% confidence interval [CI] 0.665–0.793). In 238 landmark-eligible complete cases (56 subsequent events), adding NLR change increased AUC from 0.718 to 0.801. Adding LDH yielded an AUC of 0.816, a modest, statistically non-significant increment (delta AUC 0.016; *p* = 0.094). Bootstrap-corrected calibration slopes were 0.980 for D1 and 0.957 for D2. Conclusions: NLR provided the principal dynamic contribution. D1 is emphasized as the main dynamic model; D2 remains an exploratory extension with uncertain incremental value. Unavailable laboratory collection dates and lack of external validation limit interpretation. The models and proposed thresholds require independent validation before clinical use.

## Simple Summary

Some people with advanced non-small cell lung cancer worsen or die soon after starting immunotherapy. Scans needed to measure unusually rapid tumour growth are often unavailable, and patients who die before the first scan may be overlooked. We studied 282 patients treated at one centre and defined an early event as progression or death within 90 days. A simple four-item score based on bone metastases and three routine blood tests identified patients at higher baseline risk. Among patients alive and progression-free at six weeks, change in the neutrophil-to-lymphocyte ratio provided the principal improvement in risk estimation, whereas the incremental value of lactate dehydrogenase was less certain. These findings do not diagnose hyperprogression or show who benefits from immunotherapy. The models and proposed thresholds remain exploratory and are not validated clinical decision rules. Because the score was developed and internally assessed in the same retrospective cohort, it should not guide treatment decisions until it has been tested prospectively in independent centres.

## 1. Introduction

PD-1 and PD-L1 inhibitors have changed the treatment of advanced NSCLC. Randomized trials established survival gains with pembrolizumab in PD-L1-selected disease, pembrolizumab-based chemotherapy in nonsquamous and squamous tumours, nivolumab or pembrolizumab after platinum therapy, atezolizumab after platinum therapy, and atezolizumab–bevacizumab–chemotherapy in the first-line nonsquamous setting [1,2,3,4,5,6,7,8]. Yet a clinically important minority of patients deteriorate before benefit can be assessed.

Hyperprogressive disease (HPD) describes a marked acceleration of tumour growth after immune-checkpoint inhibitor (ICI) initiation. Its biological implications are important, but the diagnosis generally requires measurable disease on pre-baseline, baseline and early on-treatment imaging. Definitions based on tumour growth rate, tumour growth kinetics or time to treatment failure identify overlapping but non-identical groups, and reported incidence varies accordingly [9,10,11,12,13,14,15,16,17]. Routine records often lack the pre-baseline scan needed for these calculations.

An early endpoint must also retain patients who die before restaging. Excluding them because radiological progression was never documented preferentially removes the most aggressive clinical courses. We therefore defined EPD90 as radiological progression or death from any cause within 90 days of the first ICI infusion. This is a pragmatic prognostic endpoint, not a surrogate definition of HPD. Fast progression has also been observed with single-agent chemotherapy, including the randomized OAK comparison of atezolizumab and docetaxel, which further argues against treating early failure as an ICI-specific phenomenon [11,18].

Published scores such as LIPI, GRIm, mGPS and EPSILoN summarize inflammatory, nutritional and clinical factors associated with prognosis [19,20,21,22,23]. LIPI combines derived NLR with LDH; mGPS combines CRP with albumin; GRIm uses NLR, albumin and LDH; and EPSILoN also incorporates performance status, smoking exposure and liver metastasis. EPS-4 combines bone metastasis with LDH, albumin and CRP in a simple additive score targeted to EPD90. These overlapping components make comparison relevant, but a different endpoint and in-cohort development do not establish an improvement over externally developed scores.

Routine blood markers may change before the first response scan. Pretreatment NLR and related inflammatory indices have been associated with outcomes across solid tumours and in ICI-treated NSCLC [24,25,26,27]. Studies of early NLR changes provide a rationale for dynamic assessment [28,29,30,31], while on-treatment LDH has also been investigated [32]. Olgun and Diker examined blood-based scores at six weeks during pembrolizumab or nivolumab treatment [33]; Matsubara et al. studied immune, inflammatory and nutritional measures in atezolizumab-treated patients [34]. These settings provide context but do not validate prediction of EPD90.

We therefore examined two linked questions: whether a simple baseline score could identify patients at higher risk of EPD90, and whether six-week changes in NLR and LDH could update that risk among patients still alive and progression-free. EPD90 was intended to capture rapid clinical deterioration, without requiring formal tumour-growth kinetics; it was not intended to diagnose HPD. We report model development with internal validation; no external validation cohort was available.

## 2. Patients and Methods

### 2.1. Study Design and Population

We conducted a retrospective single-centre cohort study of consecutive adults with pathologically confirmed advanced or unresectable NSCLC who received at least one dose of a PD-1 inhibitor, PD-L1 inhibitor or ICI-containing regimen at Akdeniz University Hospital, Antalya, Türkiye, between 1 January 2018 and 31 March 2026. The database was locked on 19 August 2026. Patients were identified from the institutional oncology treatment records; no eligible patient was deliberately sampled out.

The study was conducted in accordance with the Declaration of Helsinki and approved by the Clinical Research Ethics Committee for Medical Sciences of Akdeniz University (TBAEK-132; 26 February 2026). Individual informed consent was waived because the study used retrospectively collected, anonymized routine-care data. Reporting follows STROBE and TRIPOD [35,36].

### 2.2. Variables, Laboratory Measures and Data Quality

Demographic, clinicopathological and treatment variables included age, sex, smoking exposure, body-mass index, ECOG performance status, stage, histology, TTF-1, PD-L1 tumour proportion score, EGFR/ALK/ROS1 status, metastatic sites, treatment line, regimen and ICI agent. Baseline laboratory values were the values recorded for treatment initiation. The nominal six-week panel comprised the available on-treatment ANC, ALC and LDH values assigned to week 6 in the locked analysis extract.

The NLR was ANC/ALC, PLR was platelets/ALC and LMR was ALC/AMC. Dynamic NLR and LDH ratios were the nominal day-42 value divided by baseline; primary models used log2 ratios, so coefficients represent the effect of a doubling. The locked analysis extract contained ANC, ALC and AMC but no total white cell count (WBC). The classical derived NLR, calculated as ANC/(WBC − ANC), could therefore not be reconstructed. For an explicitly labelled approximated LIPI (aLIPI), ANC/(ALC + AMC) was used; this denominator omits other leucocyte fractions and is not interchangeable with published LIPI.

Derived variables were recalculated from raw components and reconciled with stored spreadsheet formulas. Identifiers were unique. Exact dates were checked for ordering and plausibility. Three progression dates after death, one of which also fell after the database lock, were treated as unusable progression dates; when applicable, the composite endpoint used the valid death date. Extreme but physiologically plausible laboratory values were retained. One physiologically impossible body-mass-index value was set to missing. Follow-up closed at the latest valid date among last contact, progression and death.

### 2.3. Outcomes and Time Origins

The primary baseline endpoint, EPD90, was radiological progression or death from any cause within 90 days of the first ICI infusion. It was derived from exact dates rather than rounded PFS months. Death before a valid on-treatment scan counted as an event. When progression and death were recorded on the same date, the event was assigned to progression for the event-type summary; the binary EPD90 endpoint was unaffected. Patients alive and progression-free with <90 days of follow-up were not classifiable.

Every analysis containing nominal day-42 information used an exact day-42 outcome landmark. Patients with progression or death on or before day 42 were excluded, and the dynamic endpoint was progression or death during days 43–90. This addresses the outcome-side guarantee-time bias. However, actual collection dates for nominal week-6 laboratory values were unavailable, so availability at day 42 or before a subsequent event could not be verified. Late measurements could reflect evolving deterioration and inflate apparent discrimination; outcome landmarking does not remove this measurement-timing limitation.

PFS counted radiological progression or death; OS counted death. Baseline survival analyses began at the first infusion. Analyses containing dynamic information reset the clock to day 42 or used delayed entry at day 42. Reverse Kaplan–Meier was used to estimate follow-up. Proportional hazards were tested with scaled Schoenfeld residuals; RMST differences at 6 and 12 months accompanied hazard ratios when proportionality failed.

### 2.4. Development of EPS-4 and Secondary Benchmarking

EPS-4 was developed and evaluated in this cohort as a fixed 0–4-point candidate score. Before the fitting and validation steps reported here, its four binary components and cut points were locked in the analysis code: bone metastasis, LDH above the institutional upper limit of normal (>240 U/L), albumin < 3.5 g/dL and CRP > 10 mg/L, each worth one point. The components combine a metastatic site with routinely available markers related to tissue injury, inflammation and nutritional status. Equal weighting provides a simple candidate score and does not establish equal prognostic effects. The albumin and CRP cut-points follow the clinical thresholds used in mGPS; however, EPS-4 assigns each abnormality a separate point, whereas mGPS makes the albumin contribution conditional on elevated CRP. The LDH threshold was the institutional reference limit. The exploratory univariable screen in the Supplementary Materials was descriptive and did not feed model selection. The historical process leading to the four components was not repeated within bootstrap resamples; optimism estimates therefore quantify refitting of the score-to-risk mapping but not uncertainty from component selection.

For context, GRIm, mGPS and EPSILoN followed their published definitions, while LIPI required an approximation; these scores were reconstructed and applied to the common complete-case set as secondary benchmarks. Because total WBC was not available, the LIPI comparison used an explicitly labelled approximation and is not interchangeable with published LIPI. This comparison cannot establish definitive superiority of EPS-4 over LIPI. Component definitions, availability, exact thresholds and sensitivity analyses are reported in Supplementary Tables S5a–S7.

### 2.5. Dynamic Risk Updating

The nested day-42 sequence was D0, baseline-locked EPS-4 alone; D1, D0 plus log2(NLR day 42/NLR baseline); and D2, D1 plus log2(LDH day 42/LDH baseline). No automated or stepwise selection was used. In the revised presentation, D1 is emphasized as the main dynamic model and D2 as an exploratory extension, reflecting the observed incremental results. The fitted D2 equation was logit(*p*) = −2.880 + 0.824(EPS-4) + 0.761[log2 NLR ratio] + 0.739[log2 LDH ratio]. The linear log2 LDH term preserves the originally specified simple model as an exploratory summary; evidence against linearity is reported in Section 3.8. This equation is reported for reproducibility, not for clinical deployment before external validation.

For exploratory description, we also examined whether NLR or LDH decreased from baseline and a dual-adverse state fixed for the reported analyses defined as an NLR increase ≥50% together with an LDH increase ≥25%. These categorical states and EPS-4 ≥ 2 are exploratory summaries, not validated clinical decision rules; continuous log2 ratios remained the principal dynamic terms.

The legacy seven-point dynamic score (EPS7-legacy) was reproduced only as a secondary benchmark: two points each for baseline albumin < 3.5 g/dL, baseline LDH > 250 U/L and an NLR ≥ 1.25, plus one point for an LDH ratio ≥ 1.20. Scores of 0–2, 3–4 and 5–7 were labelled low, intermediate and high. Its historical cut-points were not re-optimized, and it was not combined with EPS-4.

### 2.6. Statistical Analysis

Continuous variables are reported as median (IQR) and were compared with the Wilcoxon rank-sum test. Categorical variables are n (%) and were compared with Pearson’s chi-square or Fisher’s exact test. Absolute standardized mean differences accompany *p* values. Reporting was guided by STROBE and TRIPOD [35,36].

Discrimination was summarized by the area under the receiver operating characteristic curve (AUC) with DeLong confidence intervals and precision–recall AUC with bootstrap intervals. Accuracy was summarized by the Brier and scaled Brier scores. Score values were mapped to probabilities with one-parameter logistic models. Calibration-in-the-large, calibration slope and flexible calibration plots were examined, and clinical utility was explored with decision curves from out-of-fold predictions [37,38,39]. Comparisons with published prognostic scores were secondary: paired AUC differences were tested on identical patients with Holm adjustment, and the complete benchmarking results were reserved for the Supplementary Materials.

Nested dynamic models were compared with likelihood-ratio tests, paired ΔAUCs and changes in Brier score. Internal validation used 2000 bootstrap resamples in which each model was refitted, together with 20-times-repeated stratified cross-validation (10 folds for baseline models and 5 folds for D0–D2) [40]. Small negative optimism estimates (<0.001 AUC) for fixed one-parameter score mappings were retained rather than truncated and were interpreted as Monte Carlo variation around zero. The study size was determined by all eligible patients; no a priori sample-size target was used. D1 contained two predictor parameters and exploratory D2 contained three, with 56 events in the common landmark cohort. Shrinkage and resampling were considered alongside these descriptive counts, which do not eliminate overfitting risk [41,42].

Complete-case analysis was primary. Multiple imputation by chained equations used 30 imputations, included the outcome and Nelson–Aalen cumulative hazard as auxiliary information, and recomputed EPS-4 within each imputed dataset. Inverse-probability weighting addressed availability of nominal day-42 laboratory data. Sensitivity analyses covered landmark, endpoint, population, score-boundary and model-form assumptions; subgroups were interpreted through interaction tests.

## 3. Results

### 3.1. Cohort Flow and Early Events

Of 283 registered patients, one was event-free with <90 days of follow-up and could not be assigned an EPD90 status. The baseline cohort therefore included 282 patients, of whom 83 (29.4%) met EPD90. Under the recorded-date ordering rule, 67 events were assigned to progression and 16 to death; same-day progression and death were assigned to progression.

**Figure 1:**
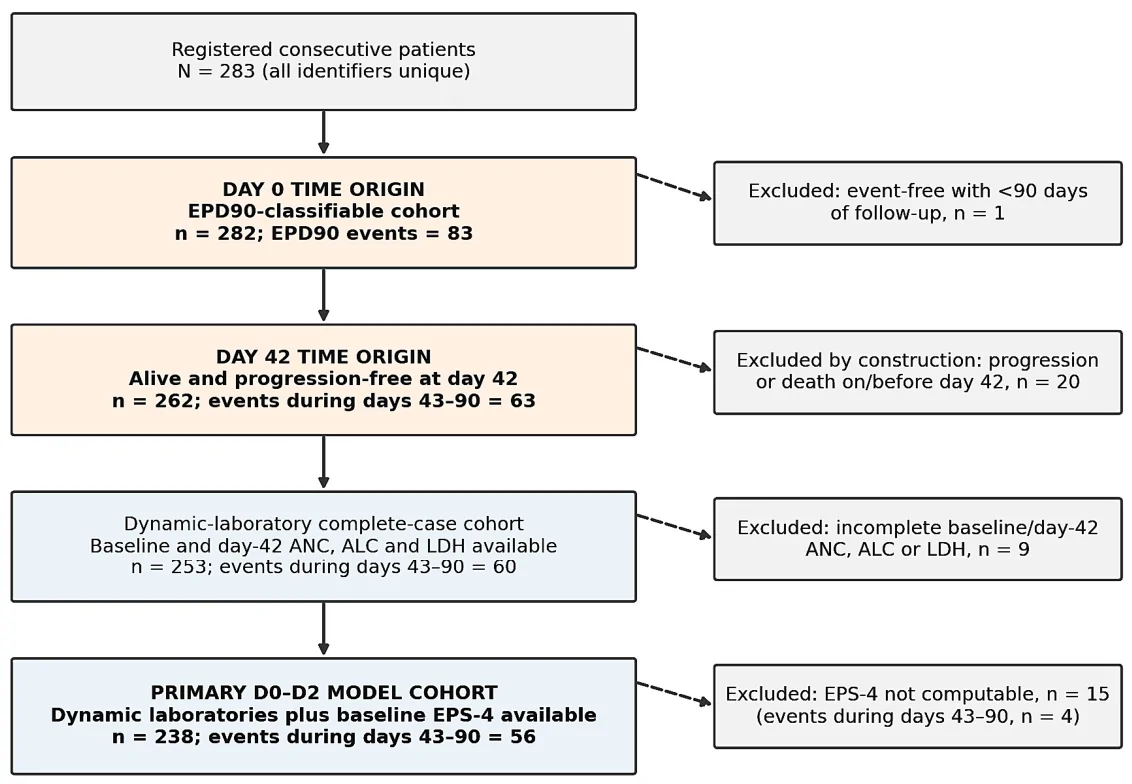
Cohort flow with two time origins. Baseline analyses begin at the first ICI infusion; every analysis containing nominal day-42 information uses an exact day-42 landmark. Patients with progression or death on or before day 42 cannot enter a landmark model. EPD90 denotes radiological progression or all-cause death within 90 days of the first infusion. Counts derive from exact dates rather than rounded PFS months. In the flow chart, orange boxes mark the two time origins, blue boxes the dynamic-laboratory analysis cohorts and grey boxes the registered cohort and the excluded patients; bold text identifies the analysis cohorts and their event counts.

The exact day-42 landmark excluded 20 patients with progression or death on or before day 42, leaving 262 patients and 63 events during days 43–90. Complete baseline and nominal day-42 ANC, ALC and LDH were available in 253 patients (60 events). Requiring complete EPS-4 components yielded the common D0–D2 cohort of 238 patients with 56 events (Figure 1).

### 3.2. Baseline Clinical Profile and the Inflammatory Phenotype

Median age was 68 years, 86.2% were men, 57.1% had adenocarcinoma, 35.5% had squamous tumours and 97.9% had stage IV disease. Treatment was predominantly second line or later (86.9%), ICI monotherapy (94.3%) and nivolumab (80.9%). EGFR/ALK/ROS1 alterations were recorded in 26 patients (9.2%), while driver status was unknown in 86 (30.5%). Liver, brain and bone metastases were present in 18.5%, 26.4% and 37.4% of patients with site information, respectively (Table 1).

Patients with EPD90 had a heavier skeletal and inflammatory burden. Bone metastasis was present in 53.8% with EPD90 versus 30.5% without EPD90 (*p* < 0.001), whereas the differences for liver and brain metastases were smaller and not statistically persuasive. The EPD90 group also had lower lymphocyte count, haemoglobin and albumin and higher CRP, LDH and NLR.

### 3.3. Divergence of NLR and LDH by the Six-Week Landmark

**Figure 2:**
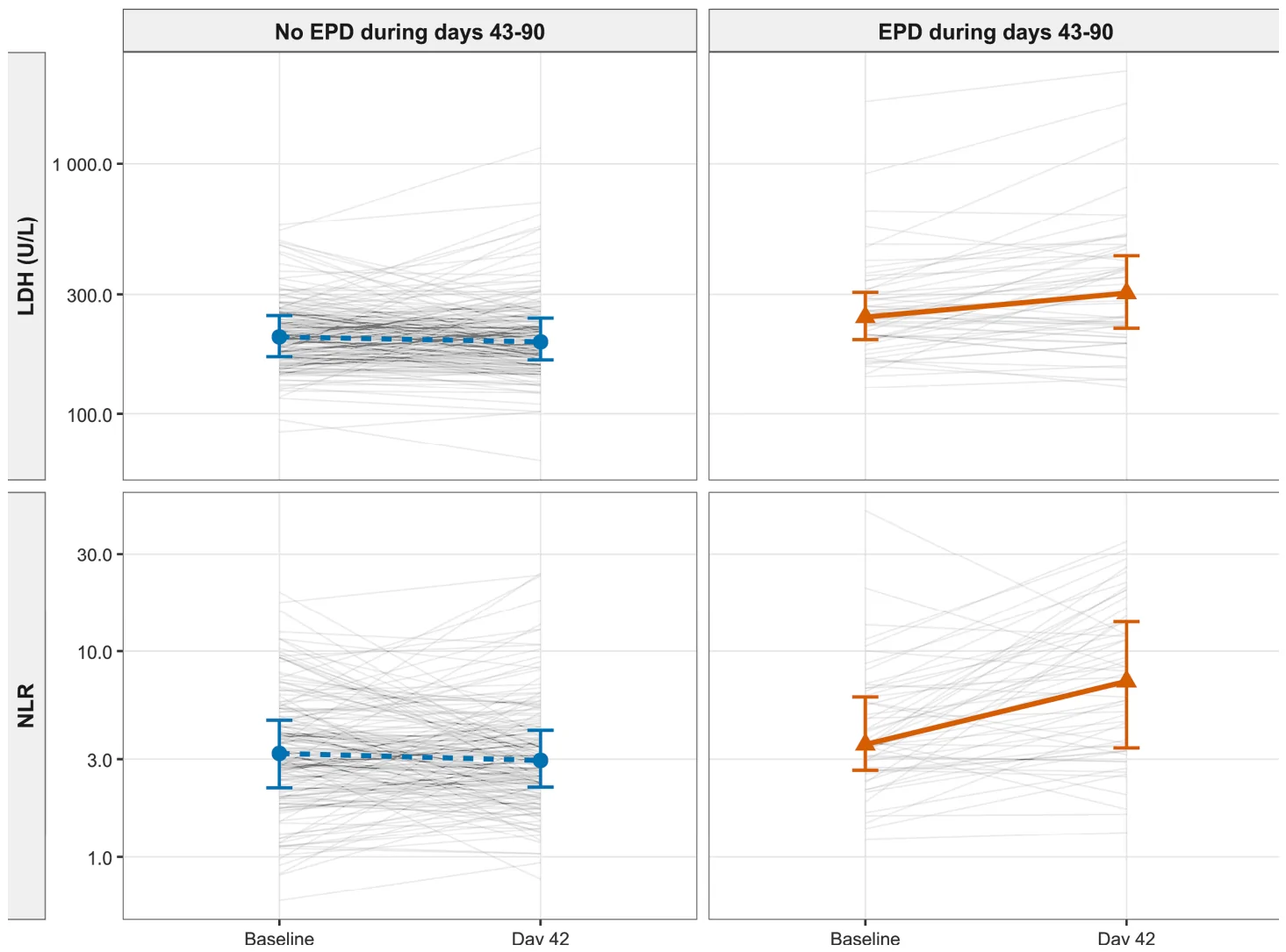
NLR and LDH trajectories from baseline to nominal day 42 in the primary D0–D2 cohort (*n* = 238; 56 events during days 43–90). Patients with progression or death on or before day 42 are excluded, so prediction begins at the exact day-42 landmark. Thin grey lines show individual patients; blue lines and bars (left panels) show medians and interquartile ranges for patients without progression or death during days 43–90, and orange lines and bars (right panels) show the corresponding values for patients with such an event. NLR is unitless, LDH is expressed in U/L and the vertical scale is logarithmic.

In the common D0-D2 landmark cohort (*n* = 238; 56 events during days 43–90), NLR and LDH were more likely to rise among patients with a subsequent event (Figure 2). An NLR decrease occurred in 111 patients and was associated with a 12.6% event rate versus 33.1% without a decrease (OR 0.29, 95% CI 0.15–0.57). LDH decrease was associated with 14.4% versus 32.5% risk (OR 0.35, 0.18–0.66). The exploratory dual-adverse pattern occurred in 25 patients; 17 (68.0%) had an event, compared with 18.3% of the remainder (OR 9.48, 3.82–23.54). All estimates above use the 238-patient common model cohort. The dynamic rows in Supplementary Table S5b use the larger laboratory-complete landmark cohort (*n* = 253; 60 events), yielding ORs of 0.35 for NLR decrease and 0.39 for LDH decrease. The differing denominators explain these estimates; neither comparison establishes a causal effect of biomarker change.

### 3.4. Baseline EPS-4 Risk Gradient and Internal Performance

In its score-specific set, the observed EPD90 rose from 9.3% at 0 EPS-4 points to 14.7%, 38.8%, 55.0% and 62.5% at 1–4 points. The corresponding univariable OR was 2.36 (95% CI 1.75–3.17) per point (*p* < 0.001). The exploratory EPS-4 ≥ 2 threshold identified 132/252 patients in the common set and had sensitivity 78.9%, specificity 59.1%, PPV 45.5% and NPV 86.7%. This threshold was fixed for descriptive analysis, was not selected to maximize Youden’s index and has not been validated as a clinical decision rule.

In the common baseline complete-case set (*n* = 252; 76 events), EPS-4 had an apparent AUC of 0.729 (95% CI 0.665–0.793), a precision–recall AUC of 0.567 and a Brier score of 0.180. Bootstrap-corrected AUC was 0.729, mean repeated-cross-validation AUC was 0.708, and pooled out-of-fold AUC was 0.659 (Supplementary Tables S8 and S9). The latter two are different summaries of internal validation; the lower pooled out-of-fold estimate indicates that the apparent AUC should not be read in isolation. The out-of-fold calibration slope was 0.958, and Brier score was 0.182 (Supplementary Table S10).

On the same 252 patients with 76 events, apparent AUCs for GRIm, mGPS, aLIPI and EPSILoN were 0.674, 0.651, 0.645 and 0.610, respectively (Supplementary Table S7). These are secondary, endpoint-specific comparisons. Published LIPI was only approximated, the comparators were developed in other settings, and EPS-4 was developed in this cohort; the numerical differences do not establish definitive superiority. Paired tests with multiplicity adjustment are provided in Table S7.

The prespecified clinical backbone—ECOG ≥2, histology, regimen and treatment line—had apparent AUC 0.542, optimism-corrected AUC 0.489 and repeated-CV AUC 0.440. Adding EPS-4 increased apparent AUC to 0.751 and corrected AUC to 0.726; paired ΔAUC was 0.210 (95% CI 0.118–0.297; *p* < 0.001), with likelihood-ratio χ^2^ = 39.7 on 1 df (*p* < 0.001). These in-cohort results suggest additional prognostic information from EPS-4 but do not establish comparative clinical utility.

### 3.5. Dynamic Discrimination and Calibration

D0 had an AUC of 0.718 (95% CI 0.647–0.789) in the common landmark cohort (*n* = 238; 56 events). Adding the log2 NLR ratio increased AUC to 0.801 (D1; ΔAUC 0.083, 95% CI 0.022–0.145; *p* = 0.006). The exploratory addition of log2 LDH ratio yielded a D2 AUC of 0.816 (0.751–0.881). Although the likelihood-ratio test indicated improved fit over D1 (*p* = 0.030), the incremental AUC difference was small and not statistically significant (ΔAUC 0.016; *p* = 0.094). These tests assess different aspects of model performance. NLR therefore supplies the principal dynamic contribution, while improved discrimination from adding LDH remains uncertain.

In D1, the OR was 2.32 (95% CI 1.63–3.31) per EPS-4 point and 2.41 (1.70–3.42) per doubling of the NLR ratio. In exploratory D2, the respective ORs were 2.28 (1.59–3.26), 2.14 (1.49–3.08) and 2.09 (1.06–4.12) for EPS-4, NLR ratio and LDH ratio (Table 2). Bootstrap-corrected AUCs for D0, D1 and D2 were 0.719, 0.796 and 0.808; mean repeated-CV AUCs were 0.707, 0.790 and 0.801. Bootstrap-corrected calibration slopes were 1.036, 0.980 and 0.957. Out-of-fold slopes were 0.932, 0.939 and 0.915. These internal calibration estimates do not establish calibration in other populations (Supplementary Tables S8–S10).

**Figure 3:**
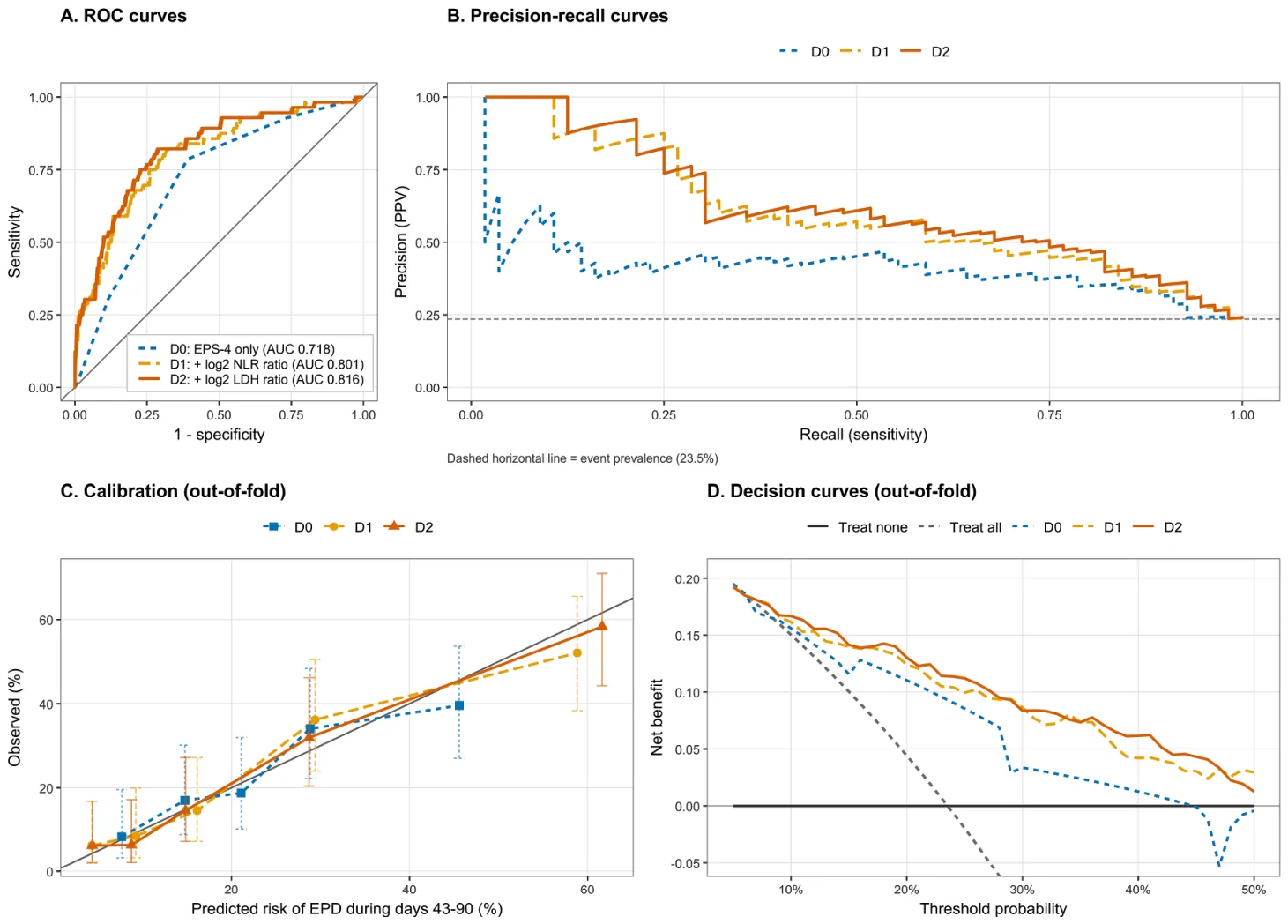
Dynamic risk updating at day 42. All panels use the same 238 patients who were alive and progression-free at the landmark; 56 experienced progression or death during days 43–90. (**A**) ROC curves; (**B**) precision–recall curves, with event prevalence (23.5%) indicated by the horizontal reference; (**C**) calibration from out-of-fold predictions; (**D**) decision curves from out-of-fold predictions. D0 is baseline-locked EPS-4, D1 is the main dynamic model adding the log2 NLR ratio and D2 is an exploratory extension adding the log2 LDH ratio; ratios are day 42 divided by baseline and effects are per doubling.

Precision–recall AUC rose from 0.425 for D0 to 0.606 for D1 and 0.626 for D2, while the Brier score improved from 0.162 to 0.137 and 0.134. At a 20% threshold, out-of-fold net benefit was 0.110, 0.124 and 0.130, respectively, compared with 0.044 for treating all (Figure 3). Decision curves describe classification consequences under assumed thresholds; they do not show that a model-triggered intervention improves outcome.

### 3.6. Exploratory Threshold Characteristics

The exploratory thresholds had different sensitivity–specificity profiles (Table 3). In the common landmark cohort, EPS-4 ≥2 had a sensitivity of 78.6% and an NPV of 90.2%. The dual-adverse state had a specificity of 95.6%, a PPV of 68.0% and LR+ 6.91, but sensitivity was only 30.4%. These are descriptive estimates from the development cohort. Neither threshold has been validated for selecting treatment, changing surveillance or prompting treatment discontinuation.

### 3.7. Exploratory Survival Analysis After the Landmark

**Figure 4:**
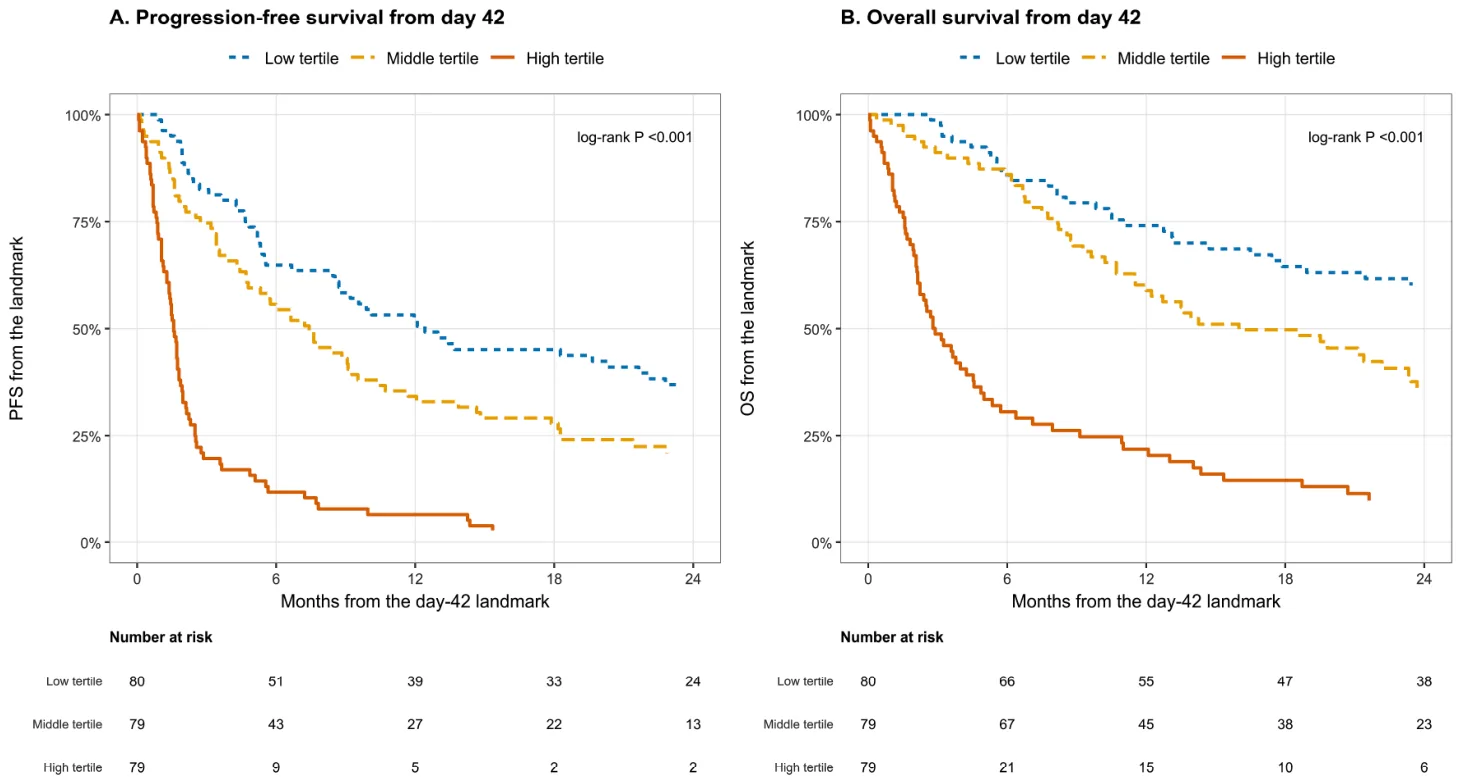
Exploratory PFS and OS from the exact day-42 landmark by tertile of D2-predicted risk in the dynamic complete-case cohort (*n* = 238; 193 PFS events and 168 deaths). (**A**) Progression-free survival from the day-42 landmark by D2-predicted-risk tertile; (**B**) overall survival from the day-42 landmark by D2-predicted-risk tertile. D2 contains EPS-4, the log2 NLR ratio and the log2 LDH ratio. Numbers at risk are displayed below each Kaplan–Meier panel. The clock starts at day 42; no patient contributes risk time before the landmark.

Exploratory D2 risk tertiles were associated with differences in subsequent PFS and OS (Figure 4; Table 4). Median PFS from day 42 was 12.4 months in the low tertile, 7.4 months in the middle and 1.6 months in the high tertile. Median OS was 28.1, 16.0 and 2.9 months, respectively. Both log-rank *p* values were <0.001. Proportional hazards were violated, driven in part by the NLR-ratio term; hazard ratios are therefore time-averaged summaries. At 12 months, high versus low risk was associated with 5.93 fewer months of PFS RMST and 5.61 fewer months of OS RMST.

### 3.8. Sensitivity, Missing-Data and Stability Analyses

Sensitivity analyses examined the 45-day grace landmark, restriction to stage IV, ICI monotherapy, second-or-later-line monotherapy, nivolumab monotherapy; exclusion of chemo-immunotherapy; alternative EPSILoN boundaries; EPD60 and EPD120; progression-only and death-only outcomes; and continuous versus prespecified categorical dynamic terms. Restricted cohorts were smaller and produced wider intervals. First-line models were not estimable because of insufficient events, limiting interpretation in that subgroup. Exact-date and stored EPD90 classifications were identical.

Thirty-fold multiple imputation, which recomputed EPS-4 within each imputed dataset, and inverse-probability weighting for day-42 laboratory availability gave estimates compatible with the complete-case results. PD-L1 adjustment among patients with known values did not materially alter EPS-4. Ridge shrinkage reduced the effective degrees of freedom of the extended adjusted model from eight to 4.3, and Firth estimates were concordant. In the existing D2 influence analysis, the LDH-ratio OR changed from 2.09 to 4.63 after excluding 16 flagged observations (Supplementary Table S15b). The association retained its direction, but its magnitude was sensitive to exclusion; these findings do not establish that extreme values are unimportant. All observations were retained in the primary analysis. The internally derived EPS7-legacy score was retained only for reproducibility: on the D2 common set, D2 AUC was 0.816 versus 0.778 for EPS7, ΔAUC 0.038 (*p* = 0.144). Because EPS7 cut-points were developed in the same cohort, this same-cohort comparison does not constitute independent validation.

The existing restricted cubic spline analysis showed evidence of nonlinearity for the log2 LDH ratio (*p* = 0.005), but not for the log2 NLR ratio (*p* = 0.779; Supplementary Table S3d). The linear LDH term in exploratory D2 is a parsimonious summary, not a verified constant effect across its range; no nonlinear model was selected or refitted for this revision.

## 4. Discussion

EPS-4 was associated with baseline EPD90 risk, and NLR change supplied the largest dynamic increment among patients alive and progression-free at day 42. We therefore emphasize D1 as the main dynamic model and retain D2 as an exploratory extension. These findings describe prognosis within a retrospective development cohort and do not establish treatment benefit or a model-guided clinical strategy.

EPD90 should not be conflated with HPD. Studies of HPD have described accelerated tumour growth and radiological or biological correlates during PD-(L)1 blockade [10,11,12,13,14,15,16,17], but this cohort lacked the serial pre-baseline imaging needed to measure growth acceleration. The observation of fast progression under docetaxel in OAK also shows that an early event endpoint is not specific to immunotherapy [18]. EPD90 is therefore best understood as a pragmatic early-risk endpoint that includes deaths before restaging.

EPS-4 combines bone metastasis and LDH with separately scored albumin and CRP abnormalities. In contrast, LIPI uses derived NLR and LDH; mGPS makes the albumin contribution conditional on elevated CRP; GRIm combines NLR, albumin and LDH; and EPSILoN includes additional clinical factors [19,20,21,22,23]. These structural differences do not establish better prediction. The secondary comparisons are limited by the EPD90 endpoint, development of EPS-4 in this cohort and incomplete reconstruction of LIPI.

The components have a clinical rationale but are nonspecific: bone metastasis records skeletal involvement, LDH may reflect tumour activity or tissue injury, and albumin and CRP reflect overlapping inflammatory and nutritional processes. Equal weighting does not establish independent mechanisms or optimal weights.

Other studies address distinct aspects of this broader biomarker context. Hakozaki et al. examined cachexia and gut microbiota during immunotherapy [43], whereas Veccia et al. evaluated inflammatory and nutritional scores in first-line ICI treatment with or without chemotherapy [44]. Mandaliya et al. investigated inflammatory indices in stage IV NSCLC [45]; Li et al. reviewed circulating immune-cell, tumour-cell and soluble biomarkers [46]; and Jiang et al. assessed peripheral blood markers during nivolumab or durvalumab monotherapy [47]. Differences in treatment, sampling and endpoints prevent these reports from serving as validation of our models.

NLR supplied the principal dynamic increment. Adding LDH improved model fit, but the AUC increment was small and statistically non-significant. Together with the existing nonlinearity and influence findings, this supports presenting D2 as exploratory. Internal calibration complements discrimination, but neither establishes reliable absolute risks in a new population.

EPS-4 ≥2 and the combined NLR/LDH threshold are exploratory descriptions of risk in this cohort. Their operating characteristics do not establish useful action thresholds, and the benefit of modifying follow-up or treatment according to either threshold has not been tested. Clinical symptoms, imaging and competing causes of laboratory abnormalities remain essential to interpretation.

NLR and LDH should not be interpreted as specific measurements of antitumour immunity. Corticosteroid exposure, intercurrent infection, systemic inflammation, tissue injury and other illnesses may alter blood counts or LDH. These factors may also be associated with poor outcomes, creating confounding, and biomarker changes may be consequences of evolving illness rather than independent antecedents. Incomplete capture of these factors prevented comprehensive adjustment.

D2 risk tertiles were associated with subsequent PFS and OS in the development cohort. Because PFS overlaps the early endpoint used to fit D2, this exploratory separation is not independent validation.

Liquid biopsy and peripheral immune-cell profiling may provide complementary information to routine inflammatory markers. Gelibter et al. examined early changes in proliferating T-cell subsets and soluble immune checkpoints during first-line immunotherapy [48]. Siringo et al. reported associations of circulating tumour-cell dynamics and cancer-associated macrophage-like cells with survival in a small retrospective NSCLC cohort receiving heterogeneous systemic treatments [49]. These studies address aspects of tumour and host biology but do not establish incremental prediction of EPD90. Such biomarkers were not evaluated in the present analysis. Prospective studies with defined sampling times and external validation would be needed to determine whether their integration improves discrimination, calibration or clinical utility beyond clinical–laboratory models.

Exact outcome dates, inclusion of early deaths and common comparison cohorts clarify the analysis. Internal validation cannot resolve uncertain laboratory timing, historical component-selection optimism or the absence of external data.

### Limitations

Missing laboratory collection dates directly limit the dynamic models. Nominal week-6 measurements could not be confirmed as available at day 42 or before an event. Late samples may reflect evolving deterioration and increase apparent discrimination. Thus, the results do not establish performance under a strictly timed day-42 sampling protocol.

Residual confounding is also important. NLR and LDH are nonspecific biomarkers: corticosteroids, intercurrent infection, systemic inflammation, tissue injury and other illnesses may alter their values and may also be associated with outcomes. These factors were incompletely captured, preventing comprehensive adjustment. Biomarker changes may reflect evolving illness rather than an independent antecedent. Progression reflected routine-care assessment rather than blinded central imaging review.

The single-centre cohort was predominantly male, and treatment was dominated by nivolumab monotherapy in the second or later line. Accrual from January 2018 to March 2026 spans changing treatment standards; small first-line and combination-treatment subgroups limit generalisability to those settings. Driver status was unknown in 30.5%, leaving residual confounding. Only 56 events supported the common dynamic models, limiting precision for nonlinear effects, interactions and subgroup estimates. EPS-4 was developed and evaluated in the same cohort; because component-selection uncertainty was not repeated in the bootstrap, corrected performance may remain optimistic. Total WBC was unavailable, so published LIPI could not be evaluated exactly.

No external validation was available. Independent temporal and multicentre prospective evaluation is required, with protocol-defined baseline and day-42 sampling, complete WBC measurements, auditable outcome assessment and adequate representation of first-line regimens. Discrimination and calibration should be assessed together. The proposed models and thresholds require validation before any model-guided clinical action can be considered.

## 5. Conclusions

EPS-4 and early NLR change were associated with EPD90 risk in this retrospective development cohort. D1 is the main dynamic model; the incremental contribution of LDH in exploratory D2 remains uncertain. These findings support further evaluation with verified sampling times and independent calibration assessment. They do not diagnose HPD, establish ICI benefit or justify clinical use of the proposed thresholds.

## Figures and Tables

**Table 1:** Selected baseline characteristics by EPD90 status.

Characteristic	Overall	No EPD90	EPD90	SMD	*p*
Age, years	68 (61–72)	68 (61–72)	66 (60–73)	0.07	0.962
Male sex	243/282 (86.2%)	171/199 (85.9%)	72/83 (86.7%)	0.02	0.856
ECOG ≥ 2	48/279 (17.2%)	32/196 (16.3%)	16/83 (19.3%)	0.08	0.551
Adenocarcinoma	161/282 (57.1%)	114/199 (57.3%)	47/83 (56.6%)	0.02 *	0.986 *
Stage IV	275/281 (97.9%)	193/199 (97.0%)	82/82 (100%)	0.25 *	0.185 *
PD-L1 TPS, %	0 (0–32)	0 (0–50)	0 (0–10)	0.16	0.139
EGFR/ALK/ROS1 positive	26/282 (9.2%)	22/199 (11.1%)	4/83 (4.8%)	0.23 *	0.254 *
Liver metastasis	49/265 (18.5%)	30/187 (16.0%)	19/78 (24.4%)	0.21	0.112
Brain metastasis	70/265 (26.4%)	46/187 (24.6%)	24/78 (30.8%)	0.14	0.299
Bone metastasis	99/265 (37.4%)	57/187 (30.5%)	42/78 (53.8%)	0.49	<0.001
Second/later line	245/282 (86.9%)	169/199 (84.9%)	76/83 (91.6%)	0.21 *	0.132 *
Lymphocytes, ×10^3^/µL	1.51 (1.06–2.10)	1.59 (1.15–2.17)	1.33 (0.80–1.75)	0.47	<0.001
Haemoglobin, g/dL	11.8 (10.7–13.1)	11.9 (10.8–13.2)	11.2 (10.1–12.7)	0.36	0.006
Albumin, g/dL	4.10 (3.80–4.40)	4.20 (3.90–4.40)	3.90 (3.30–4.20)	0.71	<0.001
CRP, mg/L	20.1 (6.0–58.0)	14.0 (3.8–43.0)	41.1 (18.0–93.0)	0.57	<0.001
LDH, U/L	213 (172–262)	203 (169–247)	247 (200–323)	0.51	<0.001
NLR	3.4 (2.4–5.4)	3.2 (2.2–4.7)	4.0 (2.9–6.9)	0.46	<0.001
EPS-4, points	2 (1–2)	1 (0–2)	2 (2–3)	0.89	<0.001

Note. Continuous variables are median (IQR); categorical variables are *n*/N (%). * SMD and p refer to the full multicategory variable. Driver status was unknown in 86/282 patients (30.5%; 59 without EPD90 and 27 with EPD90). Supplementary Table S1 describes analysis populations, and Table S2 provides variable availability and ranges. Abbreviations: CRP, C-reactive protein; ECOG, Eastern Cooperative Oncology Group performance status; EPD90, early progression or death within 90 days; EPS-4, four-item Early Progression Score; IQR, interquartile range; LDH, lactate dehydrogenase; NLR, neutrophil-to-lymphocyte ratio; PD-L1 TPS, programmed death-ligand 1 tumour proportion score; SMD, standardized mean difference.

**Table 2:** Day-42 model coefficients and performance (*n* = 238; 56 events during days 43–90).

Model	Term	OR (95% CI)	*p*	AUC (95% CI)	PR-AUC	Brier	Corrected AUC
D0	EPS-4	2.19 (1.58–3.04)	<0.001	0.718 (0.647–0.789)	0.425	0.162	0.719
D1	EPS-4	2.32 (1.63–3.31)	<0.001	0.801 (0.733–0.868)	0.606	0.137	0.796
D1	NLR ratio, per doubling	2.41 (1.70–3.42)	<0.001				
D2	EPS-4	2.28 (1.59–3.26)	<0.001	0.816 (0.751–0.881)	0.626	0.134	0.808
D2	NLR ratio, per doubling	2.14 (1.49–3.08)	<0.001				
D2	LDH ratio, per doubling	2.09 (1.06–4.12)	0.032				

Note. D0 = EPS-4 alone; D1, the main dynamic model, adds the log2 NLR ratio; exploratory D2 adds the log2 LDH ratio. AUC and Brier columns are apparent estimates; corrected AUC denotes bootstrap correction. Bootstrap-corrected calibration slopes for D0, D1 and D2 were 1.036, 0.980 and 0.957; corresponding corrected Brier scores were 0.165, 0.141 and 0.140. Supplementary Tables S8–S10 distinguish bootstrap and out-of-fold estimates.

**Table 3:** Exploratory descriptive operating characteristics of thresholds fixed for the reported analyses.

Exploratory Threshold	*N*/Events	Flagged	Sensitivity, %	Specificity, %	PPV, %	NPV, %	LR+
EPS-4 ≥ 2, baseline	252/76	132	78.9 (68.5–86.6)	59.1 (51.7–66.1)	45.5	86.7	1.93
EPS-4 ≥ 2, landmark	238/56	115	78.6 (66.2–87.3)	61.0 (53.7–67.8)	38.3	90.2	2.01
NLR +50% and LDH +25%	238/56	25	30.4 (19.9–43.3)	95.6 (91.6–97.8)	68.0	81.7	6.91

Note. EPS-4 ≥ 2 and combined NLR/LDH rises are exploratory thresholds, not validated clinical decision rules. NLR +50% and LDH +25% denotes increases of at least 50% and 25%, respectively. The displayed thresholds were not optimized in these data; full Wilson confidence intervals and comparator thresholds are in Supplementary Table S5a.

**Table 4:** Exploratory survival summaries from day 42 by D2-predicted-risk tertile.

Endpoint	Risk Tertile	Events	Median, Months (95% CI)	6-Month	12-Month	12-Month RMST Difference vs. Low
PFS	Low (*n* = 80)	53	12.4 (8.7–22.8)	64.8%	51.9%	Reference
PFS	Middle (*n* = 79)	63	7.4 (4.8–10.4)	54.4%	34.2%	−1.52 months
PFS	High (*n* = 79)	77	1.6 (1.4–1.9)	11.8%	6.5%	−5.93 months
OS	Low (*n* = 80)	42	28.1 (23.4–NR)	85.9%	74.1%	Reference
OS	Middle (*n* = 79)	58	16.0 (12.0–23.7)	86.0%	58.9%	−0.74 months
OS	High (*n* = 79)	68	2.9 (2.2–4.6)	30.6%	21.8%	−5.61 months

Note. Log-rank *p* < 0.001 for PFS and OS. RMST differences are reported because proportional hazards were not supported. NR = not reached. “Reference” in the final column denotes the referent tertile against which the restricted mean survival time (RMST) difference is calculated; it is not a bibliographic citation.

## Data Availability

The data presented in this study are available from the corresponding author upon reasonable request, subject to approval by the relevant institutional authorities and compliance with applicable ethical and data-protection requirements. The data are not publicly available because they contain individual-level clinical information and public deposition was not covered by the applicable ethics and data-protection framework.

## Supplementary Materials

The following supporting information can be downloaded at: https://www.mdpi.com/article/10.3390/curroncol33100593/s1. Supplementary Tables S1–S16 and Figures S1 provide supporting analyses. For navigation, Tables S1 and S2 describe analysis populations and variable availability; Table S5b specifies denominators for univariable estimates; Tables S6 and S7 provide score definitions and comparisons; Tables S8–S10 distinguish bootstrap, cross-validation and calibration results; and Tables S3d and S15b report nonlinearity and influence findings. Tables S11–S14 provide decision curves, sensitivity, subgroup and survival analyses, with legacy-score and reconciliation details in the relevant sub-tables and Table S16.

## Abbreviations

ALI, advanced lung cancer inflammation index; ALC, absolute lymphocyte count; ANC, absolute neutrophil count; AUC, area under the curve; CI, confidence interval; CRP, C-reactive protein; DCA, decision-curve analysis; dNLR, derived neutrophil-to-lymphocyte ratio; ECOG PS, Eastern Cooperative Oncology Group performance status; EPD90, early progression or death within 90 days; EPS-4, four-item Early Progression Score; GRIm, Gustave Roussy Immune Score; HPD, hyperprogressive disease; ICI, immune-checkpoint inhibitor; LDH, lactate dehydrogenase; LIPI, Lung Immune Prognostic Index; LMR, lymphocyte-to-monocyte ratio; mGPS, modified Glasgow Prognostic Score; NLR, neutrophil-to-lymphocyte ratio; NPV, negative predictive value; NSCLC, non-small cell lung cancer; OR, odds ratio; OS, overall survival; PD-1, programmed cell death protein 1; PD-L1, programmed death-ligand 1; PFS, progression-free survival; PLR, platelet-to-lymphocyte ratio; PPV, positive predictive value; PR-AUC, area under the precision–recall curve; RMST, restricted mean survival time; ROC, receiver-operating characteristic; WBC, white cell count.
