# Supplementary material for: Baseline and Dynamic Clinical–Laboratory Risk Models for Early Progression or Death During PD-(L)1 Blockade in Advanced NSCLC: Development and Internal Validation
Source: Curr Oncol. 2026 Sep 30;33(10):593. doi: 10.3390/curroncol33100593 (PMC13703404; doi:10.3390/curroncol33100593)
Supplement: Supplementary file 1 [file curroncol-33-00593-s001.zip › curroncol-4548671-supplementary.pdf]

Supplementary Material

Baseline and Dynamic Clinical–Laboratory Risk Models for Early Progression or Death during PD-(L)1 Blockade in Advanced NSCLC: Development and Internal Validation

Table S1a. Analysis populations

cohort	definition	n	events	event_definition
cohort_registered	all rows in the source workbook	283	-	-
cohort_baseline	EPD90 classifiable from exact dates	282	83	EPD90
week-6 labs complete (all)	ANC, ALC, LDH and NLR at day 42 all present (whole file)	270	-	-
cohort_landmark_all	EPD90 classifiable AND alive & progression-free at day 42	262	63	EPD during days 43-90
cohort_landmark_dynamic_cc	landmark-eligible AND baseline + day-42 ANC/ALC/LDH all present	253	60	EPD during days 43-90

Note. D1 is the main dynamic model and D2 an exploratory extension. The common D0-D2 cohort has 238 patients and 56 events; dynamic rows in Table S5b use the larger laboratory-complete landmark cohort of 253 patients and 60 events.

Table S1b. Cohort flow with exclusion reasons

step	n
Registered patients	283
Excluded: event-free with < 90 days follow-up (EPD90 not classifiable)	-1
Baseline EPD90 cohort	282
Excluded: progression or death on or before day 42	-20
Day-42 landmark cohort	262
Excluded: missing baseline or day-42 ANC/ALC/LDH	-9
Day-42 dynamic complete-case cohort	253

Table S1c. Follow-up (reverse Kaplan-Meier)

measure	estimate	lower	upper	n
median follow-up (reverse Kaplan-Meier, months)	33.1	30.3	37.8	282

Table S2. Data dictionary, units, ranges and missingness

variable	class	unit	n_missing_registered	pct_missing_registered	n_missing_baseline_cohort	observed_range
patient_id (IDENTIFIER - never exported)	character		0	0	0	[withheld]
age	numeric	years	0	0	0	28.00 to 91.00
sex_f	factor		0	0	0	Male; Female
ecog	numeric		3	1.1	3	0.00 to 3.00
pack_years	numeric	pack-years	8	2.8	8	0.00 to 130.00
bmi	numeric	kg/m2	1	0.4	1	15.00 to 42.30
histology3	factor		0	0	0	Adenocarcinoma; Squamous; NSCLC-NOS

variable	class	unit	n_missing_registered	pct_missing_registered	n_missing_baseline_cohort	observed_range
stage	factor		1	0.4	1	III; IV
pdl1_tps	numeric	%	63	22.3	62	0.00 to 95.00
driver_status	factor		0	0	0	Negative; Positive; Unknown
ttf1	numeric		17	6	17	0.00 to 1.00
liver_met	numeric		17	6	17	0.00 to 1.00
brain_met	numeric		17	6	17	0.00 to 1.00
bone_met	numeric		17	6	17	0.00 to 1.00
skin_met	numeric		47	16.6	47	0.00 to 1.00
prior_lines	numeric		0	0	0	0.00 to 6.00
line_first_vs_later	factor		0	0	0	First line; Second or later
chemo_io	integer		0	0	0	0.00 to 1.00
regimen_mono_vs_chemoio	factor		0	0	0	ICI monotherapy; Chemo-IO
ici_type	factor		0	0	0	Atezolizumab; Durvalumab; Ipilimumab-Nivolumab; Nivolumab; Pembrolizumab
ici_start_date	Date		0	0	0	2018-01-19 to 2026-03-30
anc0	numeric	x10^3/uL	4	1.4	4	1.43 to 24.12
alc0	numeric	x10^3/uL	4	1.4	4	0.10 to 4.69
amc0	numeric	x10^3/uL	4	1.4	4	0.06 to 2.66
platelet0	numeric	x10^3/uL	4	1.4	4	59.00 to 955.00
ldh0	numeric	U/L	4	1.4	4	85.00 to 1779.00
albumin0	numeric	g/dL	4	1.4	4	2.00 to 5.00
crp0	numeric	mg/L	4	1.4	4	0.00 to 453.00
hb0	numeric	g/dL	4	1.4	4	6.60 to 17.00
nlr0	numeric		4	1.4	4	0.61 to 48.30
adnlr0	numeric		4	1.4	4	0.51 to 17.89
plr0	numeric		4	1.4	4	37.11 to 2840.00
lmr0	numeric		4	1.4	4	0.40 to 9.57
anc42	numeric	x10^3/uL	13	4.6	13	0.86 to 48.20
alc42	numeric	x10^3/uL	13	4.6	13	0.17 to 5.09
ldh42	numeric	U/L	13	4.6	13	65.00 to 4600.00
nlr42	numeric		13	4.6	13	0.37 to 86.41
nlr_ratio	numeric		13	4.6	13	0.11 to 14.18
ldh_ratio	numeric		13	4.6	13	0.37 to 14.24
log2_nlr_ratio	numeric		13	4.6	13	-3.17 to 3.83
log2_ldh_ratio	numeric		13	4.6	13	-1.42 to 3.83
first_scan_date	Date		39	13.8	39	2018-04-24 to 2026-07-02
recist4	factor		41	14.5	41	CR; PR; SD; PD
progression_date	Date		82	29	81	2018-11-27 to 2026-09-01
death_date	Date		83	29.3	82	2019-02-18 to 2026-06-08

variable	class	unit	n_missing_registered	pct_missing_registered	n_missing_baseline_cohort	observed_range
last_followup_date	Date		0	0	0	2018-11-27 to 2026-07-30
followup_end	Date		0	0	0	2019-02-18 to 2026-09-01
event_days	numeric	days	51	18	50	3.00 to 1493.00
followup_days	numeric	days	0	0	0	10.00 to 2391.00
epd90	integer		1	0.4	0	0.00 to 1.00
epd42_90	integer		21	7.4	20	0.00 to 1.00
pfs_days	numeric	days	0	0	0	3.00 to 2102.00
pfs_event	integer		0	0	0	0.00 to 1.00
os_days	numeric	days	0	0	0	10.00 to 2391.00
os_event	integer		0	0	0	0.00 to 1.00
eps4	integer		20	7.1	20	0.00 to 4.00
grim	integer		4	1.4	4	0.00 to 3.00
mgps	integer		4	1.4	4	0.00 to 2.00
alipi	integer		4	1.4	4	0.00 to 2.00
epsilon	integer		30	10.6	30	0.00 to 4.00
eps7_legacy	integer		13	4.6	13	0.00 to 7.00

Table S3a. Exact-date EPD90 versus the workbook's stored EP/non-EP column

stored_group	non-EPD90	EPD90	NA
Belirsiz	0	0	1
EP	0	83	0
non-EP	199	0	0

Table S3b. Patient-level discordances between the two endpoint definitions

Result
No rows: no discordances or not estimable

Table S3c. Exact-date PFS/OS versus the stored month columns

check	n
stored PFS months > stored OS months	1
/exact PFS days - stored PFS months x 30.4375/ > 15 d	0
/exact OS days - stored OS months x 30.4375/ > 15 d	1

Table S3d. Restricted cubic spline tests of linearity

Note. Nonlinearity was detected for log2 LDH ratio (P = 0.005). The linear term in D2 remains an exploratory summary; this result does not confirm a constant effect across the observed range.

variable	knots	chisq	df	p	conclusion	p_fmt
log2 NLR ratio	3	0.0788244707331103	1	0.778896817048621	no evidence against linearity; linear term retained	0.779
log2 LDH ratio	3	7.83089481647067	1	0.00513606671313421	non-linearity detected; see supplementary spline figure S03	0.005

Table S4a. Week-6 laboratory availability WITHIN the landmark-eligible cohort

variable	type	n_available	n_missing	Landmark_eligible_n262	week6_available_n253	week6_MISSING_n9	smd	p	test	p_fmt	smd_fmt
Age, years	continuous	262	0	68 (61-72)	68 (61-72)	67 (59-70)	0.0504909006790869	0.857772623076299	Wilcoxon rank-sum	0.858	0.05
Sex	categorical	262	0				0.105071236178666	1	Fisher exact (simulated)	1.000	0.11
Male	level	-	-	224/262 (85.5%)	216/253 (85.4%)	8/9 (88.9%)	-	-		-	-
Female	level	-	-	38/262 (14.5%)	37/253 (14.6%)	1/9 (11.1%)	-	-		-	-
ECOG >= 2	binary	259	3	45/259 (17.4%)	42/250 (16.8%)	3/9 (33.3%)	0.388617363219249	0.192186261785586	Fisher exact	0.192	0.39
Histology	categorical	262	0				0.379562638585741	1	Fisher exact (simulated)	1.000	0.38
Adenocarcinoma	level	-	-	148/262 (56.5%)	142/253 (56.1%)	6/9 (66.7%)	-	-		-	-
Squamous	level	-	-	97/262 (37.0%)	94/253 (37.2%)	3/9 (33.3%)	-	-		-	-
NSCLC-NOS	level	-	-	17/262 (6.5%)	17/253 (6.7%)	0/9 (0.0%)	-	-		-	-
Treatment line	categorical	262	0				0.0824798505915508	1	Fisher exact (simulated)	1.000	0.08
First line	level	-	-	36/262 (13.7%)	35/253 (13.8%)	1/9 (11.1%)	-	-		-	-
Second or later	level	-	-	226/262 (86.3%)	218/253 (86.2%)	8/9 (88.9%)	-	-		-	-
Regimen	categorical	262	0				0.186429722002597	0.43800274113742	Fisher exact (simulated)	0.438	0.19
ICI monotherapy	level	-	-	246/262 (93.9%)	238/253 (94.1%)	8/9 (88.9%)	-	-		-	-
Chemo-IO	level	-	-	16/262 (6.1%)	15/253 (5.9%)	1/9 (11.1%)	-	-		-	-
Bone metastasis	binary	246	16	89/246 (36.2%)	86/238 (36.1%)	3/8 (37.5%)	0.0283155529182754	1	Fisher exact	1.000	0.03
Liver metastasis	binary	246	16	44/246 (17.9%)	42/238 (17.6%)	2/8 (25.0%)	0.180246023478625	0.636234248753375	Fisher exact	0.636	0.18
Baseline LDH, U/L	continuous	258	4	211 (172-257)	208 (172-256)	439 (213-551)	0.915952242473968	0.122757854613073	Wilcoxon rank-sum	0.123	0.92
Baseline albumin, g/dL	continuous	258	4	4.10 (3.80-4.40)	4.10 (3.80-4.40)	3.90 (3.50-4.40)	0.177437556778775	0.673155921520337	Wilcoxon rank-sum	0.673	0.18
Baseline CRP, mg/L	continuous	258	4	19.4 (5.1-51.0)	19.7 (5.8-52.0)	3.0 (1.6-30.0)	0.705259358989253	0.138926423159358	Wilcoxon rank-sum	0.139	0.71
Baseline NLR	continuous	258	4	3.3 (2.4-5.1)	3.3 (2.4-4.8)	6.9 (5.8-20.9)	1.03800745853734	0.0236091911220006	Wilcoxon rank-sum	0.024	1.04
EPS-4 score	continuous	243	19	1 (1-2)	1 (1-2)	2 (1-2)	0.141387386452315	0.700957727377729	Wilcoxon rank-sum	0.701	0.14
EPD during days 43-90	binary	262	0	63/262 (24.0%)	60/253 (23.7%)	3/9 (33.3%)	0.214225525369261	0.452620728187869	Fisher exact	0.453	0.21

Note. Restricting this comparison to landmark-eligible patients prevents early events from being misread as missing data.

Table S4b. Adjusted landmark model with ridge shrinkage

term	beta_ridge	or_ridge	beta_unpenalised	or_unpenalised	lo_unpenalised	hi_unpenalised	p_unpenalised	or_ci_unpenalised
(Intercept)	-3.1471207723656	0.0429756856865341	-4.30488143003576	0.0135024864362253	0.00250757108687158	0.0727066685825869	5.39390005204254e-07	0.01 (0.00 to 0.07)
eps4	0.622911207975033	1.86434765296668	0.887758697088264	2.42967789990732	1.65728316266881	3.56205555590849	5.41330105927567e-06	2.43 (1.66 to 3.56)
log2_nlr_ratio	0.593096908203525	1.80958386174032	0.826848756926341	2.28610331022617	1.5483483897125	3.37538268502832	3.19543962466371e-05	2.29 (1.55 to 3.38)
log2_ldh_ratio	0.70752421697523	2.02896176615116	0.873083201178467	2.39428153669138	1.16057652596672	4.93942790387409	0.0181250980414674	2.39 (1.16 to 4.94)
ecog_ge2	-0.016941890775464	0.983200816011945	-0.114737941003786	0.891599765033685	0.358499179085605	2.21743922269428	0.805038279315133	0.89 (0.36 to 2.22)
histology3Squamous	0.535151462444374	1.70770687612479	0.865910204843775	2.37716881213316	1.09081902075906	5.18044831804119	0.0293523171196545	2.38 (1.09 to 5.18)
histology3NSCLC-NOS	-0.552146578630079	0.575712670530918	-0.848174210841755	0.428196014326441	0.0640698956694161	2.86174692137938	0.38149777647618	0.43 (0.06 to 2.86)
regimen_mono_vs_chemoioChemo-IO	0.947401036585864	2.5789982196825	1.97535967255941	7.20921214335177	0.922046885692567	56.3666994968622	0.0597478357668325	7.21 (0.92 to 56.37)
line_first_vs_laterSecond or later	0.568749609259473	1.76605740843231	1.03401671277035	2.81233953806458	0.718530150553626	11.0075459899173	0.13748661685721	2.81 (0.72 to 11.01)

Table S4c. Ridge model information (events per parameter, effective df)

n	events	n_parameters	events_per_parameter	lambda_min	effective_df	note
235	56	8	7	0.0409320502670319	4.33182409168454	ridge (alpha = 0), lambda by 10-fold CV; NO stepwise selection was used

Table S5a. Exploratory descriptive operating characteristics

Rule	Analysis set	N	Events	Flagged, n	Sensitivity % (95% CI)	Specificity % (95% CI)	PPV % (95% CI)	NPV % (95% CI)	LR+	LR-
EPS-4 >= 2 (prespecified descriptive)	common cc, EPD90	252	76	132	78.9 (68.5-86.6)	59.1 (51.7-66.1)	45.5 (37.2-54.0)	86.7 (79.4-91.6)	1.93	0.36
EPS-4 >= 3 (descriptive)	common cc, EPD90	252	76	47	35.5 (25.7-46.7)	88.6 (83.1-92.5)	57.4 (43.3-70.5)	76.1 (69.8-81.4)	3.13	0.73
GRIm high risk (2-3)	common cc, EPD90	252	76	44	30.3 (21.1-41.3)	88.1 (82.4-92.1)	52.3 (37.9-66.2)	74.5 (68.2-80.0)	2.54	0.79
mGPS >= 1	common cc, EPD90	252	76	161	80.3 (70.0-87.7)	43.2 (36.1-50.6)	37.9 (30.8-45.6)	83.5 (74.6-89.7)	1.41	0.46
mGPS = 2	common cc, EPD90	252	76	38	26.3 (17.7-37.2)	89.8 (84.4-93.4)	52.6 (37.3-67.5)	73.8 (67.6-79.3)	2.57	0.82
aLIPI intermediate/poor (1-2)	common cc, EPD90	252	76	122	67.1 (55.9-76.6)	59.7 (52.3-66.6)	41.8 (33.4-50.7)	80.8 (73.2-86.6)	1.66	0.55
aLIPI poor (2)	common cc, EPD90	252	76	37	23.7 (15.5-34.4)	89.2 (83.8-93.0)	48.6 (33.4-64.1)	73.0 (66.7-78.5)	2.19	0.86
EPSILoN poor (3–5; smoking <40 pack-years)	common cc, EPD90	252	76	33	22.4 (14.5–32.9)	90.9 (85.7–94.3)	51.5 (35.2–67.5)	73.1 (66.8–78.5)	2.46	0.85
EPS-4 >= 2	landmark cc, EPD days 43-90	238	56	115	78.6 (66.2-87.3)	61.0 (53.7-67.8)	38.3 (29.9-47.4)	90.2 (83.7-94.3)	2.01	0.35
Dual adverse (NLR +50% AND LDH +25%)	landmark cc, EPD days 43-90	238	56	25	30.4 (19.9-43.3)	95.6 (91.6-97.8)	68.0 (48.4-82.8)	81.7 (76.0-86.3)	6.91	0.73
NLR did not decrease	landmark cc, EPD days 43-90	238	56	127	75.0 (62.3-84.5)	53.3 (46.1-60.4)	33.1 (25.5-41.6)	87.4 (79.9-92.3)	1.61	0.47
EPS7-legacy high (5-7)	landmark, EPS7 cc	253	60	19	23.3 (14.4-35.4)	97.4 (94.1-98.9)	73.7 (51.2-88.2)	80.3 (74.8-84.9)	9.01	0.79
EPS7-legacy intermediate/high (3-7)	landmark, EPS7 cc	253	60	78	63.3 (50.7-74.4)	79.3 (73.0-84.4)	48.7 (37.9-59.6)	87.4 (81.7-91.5)	3.06	0.46

Note. EPS-4 ≥2 and combined NLR/LDH thresholds were fixed for the reported analyses but are exploratory and are not validated clinical decision rules. Confidence intervals are Wilson intervals. The published EPSILoN smoking component (<40 pack-years) is primary; <43 pack-years is retained only as a sensitivity analysis.

Table S5b. Exploratory univariable screen with Benjamini-Hochberg q-values

Variable	n/events	OR (95% CI)	P	BH q	Outcome
Age (per year)	282/83	0.99 (0.96 to 1.02)	0.576	0.582	EPD90
ECOG >= 2	279/83	1.22 (0.63 to 2.38)	0.551	0.582	EPD90
Pack-years (per unit)	274/82	1.00 (0.98 to 1.01)	0.362	0.422	EPD90
BMI (per kg/m2)	281/83	1.04 (0.98 to 1.10)	0.237	0.368	EPD90
TTF-1 positive	265/79	0.83 (0.49 to 1.41)	0.491	0.550	EPD90
Liver metastasis	265/78	1.69 (0.88 to 3.22)	0.114	0.188	EPD90
Brain metastasis	265/78	1.36 (0.76 to 2.44)	0.300	0.420	EPD90
Bone metastasis	265/78	2.66 (1.55 to 4.58)	<0.001	0.001	EPD90
Skin metastasis	235/51	3.66 (0.22 to 59.56)	0.362	0.422	EPD90
PD-L1 TPS (per %)	220/64	0.99 (0.98 to 1.00)	0.287	0.420	EPD90
Neutrophils	278/81	1.04 (0.96 to 1.12)	0.321	0.422	EPD90
Lymphocytes	278/81	0.52 (0.35 to 0.76)	<0.001	0.002	EPD90
Monocytes	278/81	1.39 (0.70 to 2.77)	0.347	0.422	EPD90
Platelets	278/81	1.00 (1.00 to 1.00)	0.582	0.582	EPD90
Haemoglobin	278/81	0.81 (0.70 to 0.95)	0.007	0.014	EPD90
Albumin	278/81	0.25 (0.14 to 0.42)	<0.001	<0.001	EPD90
CRP	278/81	1.01 (1.01 to 1.02)	<0.001	<0.001	EPD90
LDH	278/81	1.01 (1.00 to 1.01)	<0.001	<0.001	EPD90
NLR	278/81	1.12 (1.05 to 1.20)	<0.001	0.002	EPD90
Approximated dNLR	278/81	1.21 (1.06 to 1.37)	0.003	0.008	EPD90
PLR	278/81	1.00 (1.00 to 1.00)	0.005	0.011	EPD90
LMR	278/81	0.70 (0.55 to 0.90)	0.006	0.012	EPD90
Anaemia	278/81	1.69 (0.91 to 3.12)	0.095	0.165	EPD90
EPS-4 (per point)	262/76	2.36 (1.75 to 3.17)	<0.001	<0.001	EPD90
GRIm (per point)	278/81	2.04 (1.51 to 2.76)	<0.001	<0.001	EPD90
mGPS (per point)	278/81	2.64 (1.75 to 3.96)	<0.001	<0.001	EPD90
aLIPI (per point)	278/81	2.08 (1.46 to 2.97)	<0.001	<0.001	EPD90
EPSILoN (per point)	252/76	1.53 (1.16 to 2.01)	0.003	0.007	EPD90
Age (per year)	253/60	1.00 (0.97 to 1.03)	0.892	0.948	EPD during days 43-90 (landmark)

Variable	n/events	OR (95% CI)	P	BH q	Outcome
ECOG >= 2	250/60	1.33 (0.63 to 2.80)	0.448	0.544	EPD during days 43-90 (landmark)
Pack-years (per unit)	245/59	1.00 (0.98 to 1.01)	0.537	0.630	EPD during days 43-90 (landmark)
BMI (per kg/m2)	252/60	1.02 (0.95 to 1.09)	0.660	0.748	EPD during days 43-90 (landmark)
TTF-1 positive	238/58	0.75 (0.41 to 1.37)	0.357	0.449	EPD during days 43-90 (landmark)
Liver metastasis	238/56	1.83 (0.89 to 3.79)	0.102	0.150	EPD during days 43-90 (landmark)
Brain metastasis	238/56	1.71 (0.89 to 3.28)	0.106	0.150	EPD during days 43-90 (landmark)
Bone metastasis	238/56	2.60 (1.41 to 4.79)	0.002	0.007	EPD during days 43-90 (landmark)
Skin metastasis	217/37	0.00 (0.00 to Inf)	0.988	0.997	EPD during days 43-90 (landmark)
PD-L1 TPS (per %)	197/45	0.99 (0.98 to 1.00)	0.218	0.286	EPD during days 43-90 (landmark)
Neutrophils	253/60	1.02 (0.93 to 1.11)	0.684	0.750	EPD during days 43-90 (landmark)
Lymphocytes	253/60	0.65 (0.43 to 0.98)	0.039	0.070	EPD during days 43-90 (landmark)
Monocytes	253/60	1.91 (0.90 to 4.07)	0.094	0.146	EPD during days 43-90 (landmark)
Platelets	253/60	1.00 (1.00 to 1.00)	0.997	0.997	EPD during days 43-90 (landmark)
Haemoglobin	253/60	0.81 (0.68 to 0.97)	0.021	0.042	EPD during days 43-90 (landmark)
Albumin	253/60	0.28 (0.15 to 0.50)	<0.001	<0.001	EPD during days 43-90 (landmark)
CRP	253/60	1.01 (1.00 to 1.01)	0.015	0.033	EPD during days 43-90 (landmark)
LDH	253/60	1.01 (1.00 to 1.01)	0.001	0.004	EPD during days 43-90 (landmark)
NLR	253/60	1.08 (1.00 to 1.17)	0.046	0.078	EPD during days 43-90 (landmark)
Approximated dNLR	253/60	1.11 (0.96 to 1.29)	0.142	0.193	EPD during days 43-90 (landmark)
PLR	253/60	1.00 (1.00 to 1.00)	0.083	0.134	EPD during days 43-90 (landmark)
LMR	253/60	0.71 (0.54 to 0.95)	0.021	0.042	EPD during days 43-90 (landmark)
Anaemia	253/60	2.20 (1.05 to 4.64)	0.038	0.070	EPD during days 43-90 (landmark)
EPS-4 (per point)	238/56	2.19 (1.58 to 3.04)	<0.001	<0.001	EPD during days 43-90 (landmark)
GRIm (per point)	253/60	1.76 (1.26 to 2.47)	<0.001	0.004	EPD during days 43-90 (landmark)
mGPS (per point)	253/60	2.28 (1.46 to 3.58)	<0.001	0.002	EPD during days 43-90 (landmark)
aLIPI (per point)	253/60	1.73 (1.16 to 2.56)	0.007	0.018	EPD during days 43-90 (landmark)
EPSILoN (per point)	233/57	1.47 (1.09 to 2.00)	0.012	0.030	EPD during days 43-90 (landmark)
log2 NLR ratio (per doubling)	253/60	2.31 (1.66 to 3.23)	<0.001	<0.001	EPD during days 43-90 (landmark)
log2 LDH ratio (per doubling)	253/60	3.58 (1.91 to 6.73)	<0.001	<0.001	EPD during days 43-90 (landmark)
NLR decreased	253/60	0.35 (0.19 to 0.66)	0.001	0.004	EPD during days 43-90 (landmark)
LDH decreased	253/60	0.39 (0.21 to 0.72)	0.003	0.007	EPD during days 43-90 (landmark)

Variable	n/events	OR (95% CI)	P	BH q	Outcome
Dual adverse	253/60	9.91 (4.04 to 24.32)	<0.001	<0.001	EPD during days 43-90 (landmark)
EPS7-legacy (per point)	253/60	1.94 (1.56 to 2.41)	<0.001	<0.001	EPD during days 43-90 (landmark)

Note. This screen is descriptive and did not feed the reported model-fitting steps. Dynamic rows use the laboratory-complete landmark cohort (n = 253; 60 events), rather than the common D0-D2 cohort (n = 238; 56 events). Historical component selection was not repeated within bootstrap resamples.

Table S6a. Score algorithms and exact thresholds as executed

score	component	threshold	points	note
EPS-4	Bone metastasis present	= 1	1	
EPS-4	Baseline LDH	> 240 U/L (institutional ULN)	1	
EPS-4	Baseline albumin	< 3.5 g/dL	1	
EPS-4	Baseline CRP	> 10 mg/L	1	
GRIm	Baseline NLR	> 6	1	
GRIm	Baseline albumin	< 3.5 g/dL	1	
GRIm	Baseline LDH	> 240 U/L	1	
mGPS	CRP	<= 10 mg/L -> 0 regardless of albumin	0	
mGPS	CRP raised, albumin normal	CRP > 10 and albumin >= 3.5	1	
mGPS	CRP raised, albumin low	CRP > 10 and albumin < 3.5	2	
aLIPI (approximated LIPI)	APPROXIMATED dNLR = ANC/(ALC+AMC)	> 3	1	APPROXIMATION: WBC unavailable, so the LIPI dNLR could not be computed exactly
aLIPI (approximated LIPI)	Baseline LDH	> 240 U/L	1	APPROXIMATION: WBC unavailable, so the LIPI dNLR could not be computed exactly
EPSILoN	ECOG performance status	>= 2 (ECOG 3 pooled into the adverse group)	1	
EPSILoN	Smoking burden	<40 pack-years	1	Published primary definition; <43 examined only in sensitivity analysis
EPSILoN	Liver metastasis	= 1	1	
EPSILoN	Baseline LDH	>= 400 U/L	1	
EPSILoN	Baseline NLR	>= 4	1	
EPS7-legacy (dynamic)	Baseline albumin	< 3.5 g/dL	2	Legacy score from the previous report; dynamic, landmark-only, exploratory
EPS7-legacy (dynamic)	Baseline LDH	> 250 U/L	2	Legacy score from the previous report; dynamic, landmark-only, exploratory
EPS7-legacy (dynamic)	NLR ratio (day 42 / baseline)	>= 1.25	2	Legacy score from the previous report; dynamic, landmark-only, exploratory
EPS7-legacy (dynamic)	LDH ratio (day 42 / baseline)	>= 1.20	1	Legacy score from the previous report; dynamic, landmark-only, exploratory

Table S6b. Complete-case availability of each score

score	variable	n_available_baseline_cohort	n_missing_baseline_cohort	events_in_available
EPS-4	eps4	262	20	76
GRIm	grim	278	4	81
mGPS	mgps	278	4	81
aLIPI (approximated LIPI)	alipi	278	4	81
EPSILoN	epsilon	252	30	76
ALL FIVE (common complete-case set)	-	252	30	76

Table S6c. Spearman correlations between the baseline scores

score_1	score_2	spearman_rho
EPS-4	EPS-4	1
EPS-4	GRIm	0.734704
EPS-4	mGPS	0.718732
EPS-4	aLIPI	0.628977
EPS-4	EPSILoN	0.369595
GRIm	EPS-4	0.734704
GRIm	GRIm	1
GRIm	mGPS	0.466577
GRIm	aLIPI	0.864221
GRIm	EPSILoN	0.427396
mGPS	EPS-4	0.718732
mGPS	GRIm	0.466577
mGPS	mGPS	1
mGPS	aLIPI	0.279466
mGPS	EPSILoN	0.200432
aLIPI	EPS-4	0.628977
aLIPI	GRIm	0.864221
aLIPI	mGPS	0.279466
aLIPI	aLIPI	1
aLIPI	EPSILoN	0.452729
EPSILoN	EPS-4	0.369595
EPSILoN	GRIm	0.427396
EPSILoN	mGPS	0.200432
EPSILoN	aLIPI	0.452729
EPSILoN	EPSILoN	1

Table S7a. Pairwise complete-case AUC comparisons

comparison	analysis_set	n	events	auc_1	auc_2	delta_auc	p_delong	p_holm
EPS-4 vs GRIm	pairwise complete-case set	252	76	0.728992	0.673968	0.0550239	0.0447856	0.313499
EPS-4 vs mGPS	pairwise complete-case set	252	76	0.728992	0.650942	0.0780502	0.00524609	0.0472148
EPS-4 vs aLIPI	pairwise complete-case set	252	76	0.728992	0.645372	0.0836199	0.0156284	0.125027
EPS-4 vs EPSILoN	pairwise complete-case set	252	76	0.728992	0.609674	0.119318	0.00410014	0.0410014
GRIm vs mGPS	pairwise complete-case set	252	76	0.673968	0.650942	0.0230263	0.53528	1
GRIm vs aLIPI	pairwise complete-case set	252	76	0.673968	0.645372	0.028596	0.213995	1
GRIm vs EPSILoN	pairwise complete-case set	252	76	0.673968	0.609674	0.0642943	0.116753	0.70052
mGPS vs aLIPI	pairwise complete-case set	252	76	0.650942	0.645372	0.00556968	0.901356	1
mGPS vs EPSILoN	pairwise complete-case set	252	76	0.650942	0.609674	0.0412679	0.372171	1
aLIPI vs EPSILoN	pairwise complete-case set	252	76	0.645372	0.609674	0.0356983	0.357088	1

Table S7b. Discordance between EPS-4 and the published risk groups

comparison	both_low	eps4_only	other_only	both_high	n	epd90_rate_eps4_only	epd90_rate_other_only
EPS-4 >= 2 vs GRIm high risk	117	91	3	41	252	0.406593406593407	0
EPS-4 >= 2 vs mGPS >= 1	76	15	44	117	252	0.4	0.159090909090909
EPS-4 >= 2 vs aLIPI intermediate/poor	97	33	23	99	252	0.484848484848485	0.304347826086957
EPS-4 >= 2 vs EPSILoN poor	117	102	3	30	252	0.431373	0.333333

Table S8. Bootstrap internal validation

Note. The reported bootstrap repeats model refitting but not historical EPS-4 component selection; corrected performance may remain optimistic.

Model	n/events	Apparent AUC	Optimism	Corrected AUC	Apparent Brier	Corrected Brier	Corrected slope	Validation note
EPS-4 (baseline)	252/76	0.729	0.000	0.729	0.180	0.183	1.016	the score-to-risk mapping is refitted inside every resample
GRIm (baseline)	252/76	0.674	-0.000	0.674	0.193	0.196	1.041	the score-to-risk mapping is refitted inside every resample
mGPS (baseline)	252/76	0.651	-0.000	0.651	0.196	0.199	1.065	the score-to-risk mapping is refitted inside every resample
aLIPI (baseline)	252/76	0.645	-0.001	0.646	0.198	0.201	1.074	the score-to-risk mapping is refitted inside every resample
EPSILoN (baseline)	252/76	0.610	0.001	0.608	0.203	0.206	1.161	the score-to-risk mapping is refitted inside every resample
B0: clinical backbone	259/76	0.542	0.053	0.489	0.205	0.214	0.414	the score-to-risk mapping is refitted inside every resample
B1: clinical + EPS-4	259/76	0.751	0.025	0.726	0.174	0.185	0.790	the score-to-risk mapping is refitted inside every resample
D0: EPS-4 (landmark)	238/56	0.718	-0.001	0.719	0.162	0.165	1.036	the score-to-risk mapping is refitted inside every resample
D1: + log2 NLR ratio	238/56	0.801	0.005	0.796	0.137	0.141	0.980	the score-to-risk mapping is refitted inside every resample
D2: + log2 LDH ratio	238/56	0.816	0.008	0.808	0.134	0.140	0.957	the score-to-risk mapping is refitted inside every resample
EPS7-legacy (dynamic)	253/60	0.763	-0.000	0.763	0.146	0.148	1.016	PARTIAL correction only: the bootstrap refits the score-to-risk mapping but does NOT re-derive the data-driven cut-points, so this value is still optimistic

Table S9. Repeated stratified cross-validation

Note. Baseline models used 10 folds and D0-D2 used 5 folds, each repeated 20 times. Mean cross-validation AUC and pooled out-of-fold AUC are distinct summaries and are labelled separately.

Model	n/events	Folds × repeats	Mean CV AUC (empirical range)	Pooled OOF AUC	OOF Brier	OOF calibration slope	OOF intercept	Formula
EPS-4 (baseline)	252/76	10 × 20	0.708 (0.696–0.717)	0.659	0.182	0.958	-0.002	epd90 ~ eps4
GRIm (baseline)	252/76	10 × 20	0.646 (0.631–0.666)	0.568	0.194	0.943	-0.001	epd90 ~ grim
mGPS (baseline)	252/76	10 × 20	0.626 (0.607–0.647)	0.564	0.197	0.938	-0.001	epd90 ~ mgps
aLIPI (baseline)	252/76	10 × 20	0.614 (0.595–0.627)	0.527	0.199	0.936	-0.001	epd90 ~ alipi
EPSILoN (baseline)	252/76	10 × 20	0.592 (0.579–0.607)	0.549	0.204	0.895	-0.002	epd90 ~ epsilon
B0: clinical backbone	259/76	10 × 20	0.440 (0.417–0.456)	0.358	0.212	-0.197	-0.001	epd90 ~ ecog_ge2 + histology3 + regimen_mono_vs_chemoio + line_first_vs_later
B1: clinical + EPS-4	259/76	10 × 20	0.717 (0.706–0.723)	0.705	0.184	0.807	0.001	epd90 ~ ecog_ge2 + histology3 + regimen_mono_vs_chemoio + line_first_vs_later + eps4
D0: EPS-4 (landmark)	238/56	5 × 20	0.707 (0.690–0.722)	0.675	0.164	0.932	-0.005	epd42_90 ~ eps4
D1: + log2 NLR ratio	238/56	5 × 20	0.790 (0.778–0.797)	0.791	0.140	0.939	-0.007	epd42_90 ~ eps4 + log2_nlr_ratio
D2: + log2 LDH ratio	238/56	5 × 20	0.801 (0.791–0.809)	0.800	0.139	0.915	-0.014	epd42_90 ~ eps4 + log2_nlr_ratio + log2_ldh_ratio
EPS7-legacy (dynamic)	253/60	10 × 20	0.742 (0.735–0.751)	0.704	0.147	0.953	-0.000	epd42_90 ~ eps7_legacy

Table S10a. Calibration statistics

Note. Calibration should be interpreted alongside discrimination and Brier scores. All validation assessments are internal and do not establish calibration in other populations.

Model	Prediction	n/events	Calibration intercept	Calibration slope	Brier	Scaled Brier	Note
EPS-4 (baseline)	apparent (in-sample)	252/76	-1.176e-16	1	0.180	0.145	intercept 0 and slope 1 are forced by same-data fitting; calibration slope and plot are primary
EPS-4 (baseline)	out-of-fold	252/76	-0.00209	0.9577	0.182	0.138	out-of-fold prediction; calibration slope and plot are primary
GRIm (baseline)	apparent (in-sample)	252/76	-3.673e-16	1	0.193	0.085	intercept 0 and slope 1 are forced by same-data fitting; calibration slope and plot are primary

Model	Prediction	n/events	Calibration intercept	Calibration slope	Brier	Scaled Brier	Note
GRIm (baseline)	out-of-fold	252/76	-0.001166	0.9426	0.194	0.078	out-of-fold prediction; calibration slope and plot are primary
mGPS (baseline)	apparent (in-sample)	252/76	1.801e-17	1	0.196	0.070	intercept 0 and slope 1 are forced by same-data fitting; calibration slope and plot are primary
mGPS (baseline)	out-of-fold	252/76	-0.0005153	0.938	0.197	0.063	out-of-fold prediction; calibration slope and plot are primary
aLIPI (baseline)	apparent (in-sample)	252/76	-5.358e-17	1	0.198	0.061	intercept 0 and slope 1 are forced by same-data fitting; calibration slope and plot are primary
aLIPI (baseline)	out-of-fold	252/76	-0.0008515	0.936	0.199	0.054	out-of-fold prediction; calibration slope and plot are primary
EPSILoN (baseline)	apparent (in-sample)	252/76	-1.043e-16	1	0.203	0.038	intercept 0 and slope 1 are forced by same-data fitting; calibration slope and plot are primary
EPSILoN (baseline)	out-of-fold	252/76	-0.00178	0.8954	0.204	0.031	out-of-fold prediction; calibration slope and plot are primary
B1: clinical + EPS-4	apparent (in-sample)	259/76	-2.016e-09	1	0.174	0.159	intercept 0 and slope 1 are FORCED by fitting and evaluating in the same data; no evidential value
B1: clinical + EPS-4	out-of-fold	259/76	0.0005768	0.807	0.184	0.113	out-of-fold prediction; this is the interpretable calibration
D0: EPS-4 (landmark)	apparent (in-sample)	238/56	-3.9e-10	1	0.162	0.101	intercept 0 and slope 1 are FORCED by fitting and evaluating in the same data; no evidential value
D0: EPS-4 (landmark)	out-of-fold	238/56	-0.005412	0.9324	0.164	0.091	out-of-fold prediction; this is the interpretable calibration
D1: + log2 NLR ratio	apparent (in-sample)	238/56	4.135e-11	1	0.137	0.238	intercept 0 and slope 1 are FORCED by fitting and evaluating in the same data; no evidential value
D1: + log2 NLR ratio	out-of-fold	238/56	-0.007443	0.9393	0.140	0.221	out-of-fold prediction; this is the interpretable calibration

Model	Prediction	n/events	Calibration intercept	Calibration slope	Brier	Scaled Brier	Note
D2: + log2 LDH ratio	apparent (in-sample)	238/56	5.593e-11	1	0.134	0.255	intercept 0 and slope 1 are FORCED by fitting and evaluating in the same data; no evidential value
D2: + log2 LDH ratio	out-of-fold	238/56	-0.01391	0.9147	0.139	0.227	out-of-fold prediction; this is the interpretable calibration

Table S10b. Binned observed versus predicted risk

model	prediction	bin	n	mean_predicted	observed	obs_lo	obs_hi
EPS-4 (baseline)	apparent (in-sample)	0.090	53	0.090	0.094	0.041	0.203
EPS-4 (baseline)	apparent (in-sample)	0.190	67	0.190	0.164	0.094	0.271
EPS-4 (baseline)	apparent (in-sample)	0.569	40	0.569	0.550	0.398	0.693
EPS-4 (baseline)	apparent (in-sample)	0.358	85	0.358	0.388	0.292	0.495
EPS-4 (baseline)	apparent (in-sample)	0.757	7	0.757	0.714	0.359	0.918
EPS-4 (baseline)	out-of-fold	(0.081, 0.0937]	51	0.090	0.098	0.043	0.210
EPS-4 (baseline)	out-of-fold	(0.0937, 0.191]	50	0.186	0.220	0.128	0.352
EPS-4 (baseline)	out-of-fold	(0.361, 0.781]	51	0.580	0.529	0.395	0.659
EPS-4 (baseline)	out-of-fold	(0.191, 0.358]	50	0.294	0.400	0.276	0.538
EPS-4 (baseline)	out-of-fold	(0.358, 0.361]	50	0.359	0.260	0.159	0.396
GRIm (baseline)	apparent (in-sample)	0.192	123	0.192	0.171	0.114	0.247
GRIm (baseline)	apparent (in-sample)	0.327	85	0.327	0.376	0.281	0.483
GRIm (baseline)	apparent (in-sample)	0.499	29	0.499	0.483	0.314	0.656
GRIm (baseline)	apparent (in-sample)	0.671	15	0.671	0.600	0.357	0.802
GRIm (baseline)	out-of-fold	(0.191, 0.194]	50	0.192	0.040	0.011	0.135
GRIm (baseline)	out-of-fold	(0.194, 0.327]	50	0.268	0.340	0.224	0.478
GRIm (baseline)	out-of-fold	(0.329, 0.694]	51	0.529	0.471	0.341	0.605
GRIm (baseline)	out-of-fold	(0.327, 0.329]	50	0.328	0.280	0.175	0.417
GRIm (baseline)	out-of-fold	(0.186, 0.191]	51	0.190	0.373	0.253	0.510
mGPS (baseline)	apparent (in-sample)	0.169	91	0.169	0.165	0.103	0.254
mGPS (baseline)	apparent (in-sample)	0.327	123	0.327	0.333	0.256	0.421
mGPS (baseline)	apparent (in-sample)	0.537	38	0.537	0.526	0.373	0.675
mGPS (baseline)	out-of-fold	(0.17, 0.326]	50	0.202	0.120	0.056	0.238
mGPS (baseline)	out-of-fold	(0.326, 0.327]	50	0.326	0.380	0.259	0.518
mGPS (baseline)	out-of-fold	(0.327, 0.328]	50	0.327	0.280	0.175	0.417
mGPS (baseline)	out-of-fold	(0.328, 0.554]	51	0.484	0.431	0.305	0.567
mGPS (baseline)	out-of-fold	(0.162, 0.17]	51	0.167	0.294	0.187	0.430
aLIPI (baseline)	apparent (in-sample)	0.205	130	0.205	0.192	0.134	0.268
aLIPI (baseline)	apparent (in-sample)	0.350	85	0.350	0.388	0.292	0.495
aLIPI (baseline)	apparent (in-sample)	0.530	37	0.530	0.486	0.334	0.641
aLIPI (baseline)	out-of-fold	(0.204, 0.206]	50	0.205	0.060	0.021	0.162
aLIPI (baseline)	out-of-fold	(0.35, 0.351]	50	0.350	0.300	0.191	0.438
aLIPI (baseline)	out-of-fold	(0.206, 0.35]	50	0.267	0.280	0.175	0.417
aLIPI (baseline)	out-of-fold	(0.198, 0.204]	51	0.202	0.431	0.305	0.567
aLIPI (baseline)	out-of-fold	(0.351, 0.543]	51	0.483	0.431	0.305	0.567
EPSILoN (baseline)	apparent (in-sample)	0.270	104	0.270	0.279	0.202	0.372
EPSILoN (baseline)	apparent (in-sample)	0.463	29	0.463	0.517	0.344	0.686
EPSILoN (baseline)	apparent (in-sample)	0.361	59	0.361	0.322	0.217	0.449
EPSILoN (baseline)	apparent (in-sample)	0.195	56	0.195	0.196	0.113	0.318
EPSILoN (baseline)	apparent (in-sample)	0.568	4	0.568	0.500	0.150	0.850
EPSILoN (baseline)	out-of-fold	(0.2, 0.27]	50	0.262	0.360	0.241	0.499

model	prediction	bin	n	mean_predicted	observed	obs_lo	obs_hi
EPSILoN (baseline)	out-of-fold	(0.362, 0.595]	51	0.437	0.392	0.270	0.529
EPSILoN (baseline)	out-of-fold	(0.271, 0.362]	50	0.344	0.320	0.208	0.458
EPSILoN (baseline)	out-of-fold	(0.186, 0.2]	51	0.195	0.216	0.125	0.346
EPSILoN (baseline)	out-of-fold	(0.27, 0.271]	50	0.270	0.220	0.128	0.352
B1: clinical + EPS-4	apparent (in-sample)	[0.0168,0.108]	58	0.084	0.086	0.037	0.186
B1: clinical + EPS-4	apparent (in-sample)	(0.108,0.183]	47	0.169	0.128	0.060	0.252
B1: clinical + EPS-4	apparent (in-sample)	(0.183,0.344]	52	0.267	0.308	0.199	0.443
B1: clinical + EPS-4	apparent (in-sample)	(0.344,0.423]	57	0.382	0.421	0.302	0.550
B1: clinical + EPS-4	apparent (in-sample)	(0.423,0.815]	45	0.612	0.556	0.412	0.691
B1: clinical + EPS-4	out-of-fold	[0.0165,0.111]	52	0.080	0.115	0.054	0.230
B1: clinical + EPS-4	out-of-fold	(0.111,0.188]	52	0.162	0.135	0.067	0.253
B1: clinical + EPS-4	out-of-fold	(0.188,0.349]	51	0.256	0.353	0.236	0.490
B1: clinical + EPS-4	out-of-fold	(0.349,0.428]	52	0.375	0.385	0.265	0.520
B1: clinical + EPS-4	out-of-fold	(0.428,0.812]	52	0.593	0.481	0.351	0.613
D0: EPS-4 (landmark)	apparent (in-sample)	0.077	52	0.077	0.077	0.030	0.182
D0: EPS-4 (landmark)	apparent (in-sample)	0.155	71	0.155	0.113	0.058	0.207
D0: EPS-4 (landmark)	apparent (in-sample)	0.288	77	0.288	0.351	0.253	0.462
D0: EPS-4 (landmark)	apparent (in-sample)	0.469	33	0.469	0.455	0.298	0.620
D0: EPS-4 (landmark)	apparent (in-sample)	0.660	5	0.660	0.400	0.118	0.769
D0: EPS-4 (landmark)	out-of-fold	[0.0694,0.0809]	48	0.077	0.083	0.033	0.196
D0: EPS-4 (landmark)	out-of-fold	(0.0809,0.156]	47	0.148	0.170	0.089	0.301
D0: EPS-4 (landmark)	out-of-fold	(0.156,0.287]	48	0.211	0.188	0.102	0.319
D0: EPS-4 (landmark)	out-of-fold	(0.287,0.29]	47	0.288	0.340	0.222	0.483
D0: EPS-4 (landmark)	out-of-fold	(0.29,0.701]	48	0.456	0.396	0.270	0.537
D1: + log2 NLR ratio	apparent (in-sample)	[0.00613,0.0633]	48	0.044	0.062	0.021	0.168
D1: + log2 NLR ratio	apparent (in-sample)	(0.0633,0.119]	47	0.093	0.085	0.034	0.199
D1: + log2 NLR ratio	apparent (in-sample)	(0.119,0.205]	48	0.162	0.125	0.059	0.247
D1: + log2 NLR ratio	apparent (in-sample)	(0.205,0.393]	47	0.292	0.340	0.222	0.483
D1: + log2 NLR ratio	apparent (in-sample)	(0.393,0.89]	48	0.585	0.562	0.423	0.693
D1: + log2 NLR ratio	out-of-fold	[0.00621,0.064]	48	0.043	0.062	0.021	0.168
D1: + log2 NLR ratio	out-of-fold	(0.064,0.119]	47	0.093	0.085	0.034	0.199
D1: + log2 NLR ratio	out-of-fold	(0.119,0.203]	48	0.162	0.146	0.072	0.272
D1: + log2 NLR ratio	out-of-fold	(0.203,0.391]	47	0.294	0.362	0.240	0.505
D1: + log2 NLR ratio	out-of-fold	(0.391,0.889]	48	0.588	0.521	0.383	0.655
D2: + log2 LDH ratio	apparent (in-sample)	[0.00594,0.0634]	48	0.044	0.062	0.021	0.168
D2: + log2 LDH ratio	apparent (in-sample)	(0.0634,0.107]	47	0.088	0.043	0.012	0.142
D2: + log2 LDH ratio	apparent (in-sample)	(0.107,0.201]	48	0.148	0.125	0.059	0.247
D2: + log2 LDH ratio	apparent (in-sample)	(0.201,0.399]	47	0.286	0.340	0.222	0.483
D2: + log2 LDH ratio	apparent (in-sample)	(0.399,0.904]	48	0.609	0.604	0.463	0.730
D2: + log2 LDH ratio	out-of-fold	[0.00603,0.0626]	48	0.044	0.062	0.021	0.168
D2: + log2 LDH ratio	out-of-fold	(0.0626,0.106]	47	0.088	0.064	0.022	0.172
D2: + log2 LDH ratio	out-of-fold	(0.106,0.201]	48	0.149	0.146	0.072	0.272
D2: + log2 LDH ratio	out-of-fold	(0.201,0.401]	47	0.287	0.319	0.204	0.462
D2: + log2 LDH ratio	out-of-fold	(0.401,0.905]	48	0.616	0.583	0.443	0.712

Table S10c. EPS7-legacy performance

model	n	events	prevalence	auc	auc_lo	auc_hi	pr_auc	pr_auc_lo	pr_auc_hi	brier	brier_scaled	calib_intercept	calib_slope	auc_ci
EPS7-legacy (dynamic, per point)	253	60	0.237154150197628	0.762564766839378	0.69084646386896	0.834283069809796	0.558321391580211	0.408330926842828	0.668574808531669	0.1455461884844	0.195486530336965	-1.62300870531649e-08	0.999999999999998	0.763 (0.691 to 0.834)

Table S10d. EPS7-legacy event rate by point

points	n	events	pct	ci
0	77	7	9.09090909090909	4.5-17.6
1	17	1	5.88235294117647	1.0-27.0
2	81	14	17.2839506172839	10.6-26.9
3	36	12	33.3333333333333	20.2-49.7
4	23	12	52.1739130434783	33.0-70.8
5	16	12	75	50.5-89.8
6	1	1	100	20.7-100.0
7	2	1	50	9.5-90.5

Table S10e. EPS7-legacy event rate by risk group

group	n	events	pct	ci
Low (0-2)	175	22	12.5714285714286	8.5-18.3
Intermediate (3-4)	59	24	40.6779661016949	29.1-53.4
High (5-7)	19	14	73.6842105263158	51.2-88.2

Table S10f. Dynamic model D2 versus EPS7-legacy

comparison	n	events	auc_D2	auc_EPS7	delta_auc	p_delong	note
D2 (EPS-4 + log2 NLR ratio + log2 LDH ratio) vs EPS7-legacy	238	56	0.816326530612245	0.777864992150706	0.0384615384615385	0.143976897773538	both apparent; EPS7-legacy was derived in this cohort so its AUC is optimistic

Table S10g. Net reclassification improvement (supplementary)

comparison	metric	value	warning
D2 vs D0 (out-of-fold predictions)	nri_events	0.25	SUPPLEMENTARY ONLY. Continuous NRI has no interpretable null and is not primary evidence; the primary comparisons are delta-AUC, delta-Brier, calibration and decision curves.
D2 vs D0 (out-of-fold predictions)	nri_nonevents	0.417582417582418	SUPPLEMENTARY ONLY. Continuous NRI has no interpretable null and is not primary evidence; the primary comparisons are delta-AUC, delta-Brier, calibration and decision curves.
D2 vs D0 (out-of-fold predictions)	nri_total	0.667582417582418	SUPPLEMENTARY ONLY. Continuous NRI has no interpretable null and is not primary evidence; the primary comparisons are delta-AUC, delta-Brier, calibration and decision curves.
D2 vs D0 (out-of-fold predictions)	categorical_nri_events	0.25	SUPPLEMENTARY ONLY. Continuous NRI has no interpretable null and is not primary evidence; the primary comparisons are delta-AUC, delta-Brier, calibration and decision curves.
D2 vs D0 (out-of-fold predictions)	categorical_nri_nonevents	0.175824175824176	SUPPLEMENTARY ONLY. Continuous NRI has no interpretable null and is not primary evidence; the primary comparisons are delta-AUC, delta-Brier, calibration and decision curves.
D2 vs D0 (out-of-fold predictions)	categorical_nri_total	0.425824175824176	SUPPLEMENTARY ONLY. Continuous NRI has no interpretable null and is not primary evidence; the primary comparisons are delta-AUC, delta-Brier, calibration and decision curves.

Note. Supplementary only. Continuous NRI has no interpretable null; the primary incremental evidence is the change in AUC, the change in Brier score, calibration and net benefit.

Table S11a. Decision-curve net benefit grid, baseline scores

threshold	treat_none	treat_all	EPS-4 (baseline)	GRIm (baseline)	mGPS (baseline)	aLIPI (baseline)	EPSILoN (baseline)	analysis_set
0.050	0	0.265	0.265	0.265	0.265	0.265	0.265	common complete-case set (out-of-fold predictions)
0.060	0	0.257	0.257	0.257	0.257	0.257	0.257	common complete-case set (out-of-fold predictions)
0.070	0	0.249	0.249	0.249	0.249	0.249	0.249	common complete-case set (out-of-fold predictions)
0.080	0	0.241	0.241	0.241	0.241	0.241	0.241	common complete-case set (out-of-fold predictions)
0.090	0	0.233	0.219	0.233	0.233	0.233	0.233	common complete-case set (out-of-fold predictions)
0.100	0	0.224	0.225	0.224	0.224	0.224	0.224	common complete-case set (out-of-fold predictions)
0.110	0	0.215	0.219	0.215	0.215	0.215	0.215	common complete-case set (out-of-fold predictions)
0.120	0	0.206	0.212	0.206	0.206	0.206	0.206	common complete-case set (out-of-fold predictions)
0.130	0	0.197	0.206	0.197	0.197	0.197	0.197	common complete-case set (out-of-fold predictions)

threshold	treat_none	treat_all	EPS-4 (baseline)	GRIm (baseline)	mGPS (baseline)	aLIPI (baseline)	EPSILoN (baseline)	analysis_set
0.140	0	0.188	0.199	0.188	0.188	0.188	0.188	common complete-case set (out-of-fold predictions)
0.150	0	0.178	0.192	0.178	0.178	0.178	0.178	common complete-case set (out-of-fold predictions)
0.160	0	0.169	0.185	0.169	0.169	0.169	0.169	common complete-case set (out-of-fold predictions)
0.170	0	0.159	0.178	0.159	0.133	0.159	0.159	common complete-case set (out-of-fold predictions)
0.180	0	0.148	0.170	0.148	0.155	0.148	0.148	common complete-case set (out-of-fold predictions)
0.190	0	0.138	0.145	0.085	0.149	0.138	0.122	common complete-case set (out-of-fold predictions)
0.200	0	0.127	0.167	0.145	0.143	0.119	0.123	common complete-case set (out-of-fold predictions)
0.210	0	0.116	0.162	0.140	0.137	0.127	0.120	common complete-case set (out-of-fold predictions)
0.220	0	0.105	0.158	0.135	0.130	0.123	0.111	common complete-case set (out-of-fold predictions)
0.230	0	0.093	0.153	0.131	0.124	0.118	0.103	common complete-case set (out-of-fold predictions)
0.240	0	0.081	0.148	0.126	0.117	0.113	0.094	common complete-case set (out-of-fold predictions)
0.250	0	0.069	0.143	0.120	0.110	0.108	0.085	common complete-case set (out-of-fold predictions)
0.260	0	0.056	0.138	0.115	0.103	0.103	0.075	common complete-case set (out-of-fold predictions)
0.270	0	0.043	0.132	0.110	0.095	0.098	0.044	common complete-case set (out-of-fold predictions)
0.280	0	0.030	0.127	0.104	0.088	0.093	0.056	common complete-case set (out-of-fold predictions)
0.290	0	0.016	0.121	0.098	0.080	0.087	0.052	common complete-case set (out-of-fold predictions)
0.300	0	0.002	0.116	0.092	0.072	0.082	0.048	common complete-case set (out-of-fold predictions)
0.310	0	-0.012	0.110	0.086	0.064	0.076	0.043	common complete-case set (out-of-fold predictions)
0.320	0	-0.027	0.104	0.080	0.055	0.070	0.038	common complete-case set (out-of-fold predictions)
0.330	0	-0.042	0.097	0.050	0.044	0.064	0.033	common complete-case set (out-of-fold predictions)
0.340	0	-0.058	0.091	0.048	0.043	0.057	0.028	common complete-case set (out-of-fold predictions)
0.350	0	-0.074	0.084	0.046	0.041	0.005	0.023	common complete-case set (out-of-fold predictions)
0.360	0	-0.091	0.046	0.044	0.039	0.029	-0.007	common complete-case set (out-of-fold predictions)
0.370	0	-0.109	0.061	0.042	0.037	0.027	0.030	common complete-case set (out-of-fold predictions)
0.380	0	-0.126	0.058	0.040	0.036	0.025	0.029	common complete-case set (out-of-fold predictions)
0.390	0	-0.145	0.056	0.038	0.034	0.023	0.027	common complete-case set (out-of-fold predictions)
0.400	0	-0.164	0.054	0.036	0.032	0.021	0.025	common complete-case set (out-of-fold predictions)
0.410	0	-0.184	0.052	0.033	0.030	0.019	0.023	common complete-case set (out-of-fold predictions)
0.420	0	-0.204	0.050	0.031	0.028	0.017	0.021	common complete-case set (out-of-fold predictions)
0.430	0	-0.225	0.047	0.028	0.025	0.015	0.020	common complete-case set (out-of-fold predictions)
0.440	0	-0.247	0.045	0.026	0.023	0.012	0.018	common complete-case set (out-of-fold predictions)
0.450	0	-0.270	0.042	0.023	0.021	0.010	0.016	common complete-case set (out-of-fold predictions)
0.460	0	-0.293	0.040	0.020	0.019	0.007	-0.026	common complete-case set (out-of-fold predictions)
0.470	0	-0.318	0.037	0.017	0.016	0.005	-0.024	common complete-case set (out-of-fold predictions)
0.480	0	-0.343	0.034	0.014	0.013	0.002	6.11e-04	common complete-case set (out-of-fold predictions)
0.490	0	-0.369	0.031	0.011	0.011	-0.001	3.11e-04	common complete-case set (out-of-fold predictions)
0.500	0	-0.397	0.028	-0.040	0.008	-0.004	0	common complete-case set (out-of-fold predictions)

Table S11b. Decision-curve net benefit grid, landmark models

threshold	treat_none	treat_all	D0: EPS-4 (landmark)	D1: + log2 NLR ratio	D2: + log2 LDH ratio	analysis_set
0.050	0	0.195	0.195	0.193	0.192	day-42 landmark complete-case set (out-of-fold predictions)
0.060	0	0.186	0.186	0.185	0.185	day-42 landmark complete-case set (out-of-fold predictions)
0.070	0	0.178	0.170	0.181	0.181	day-42 landmark complete-case set (out-of-fold predictions)
0.080	0	0.169	0.166	0.178	0.177	day-42 landmark complete-case set (out-of-fold predictions)
0.090	0	0.160	0.163	0.166	0.167	day-42 landmark complete-case set (out-of-fold predictions)
0.100	0	0.150	0.156	0.162	0.167	day-42 landmark complete-case set (out-of-fold predictions)
0.110	0	0.141	0.149	0.153	0.163	day-42 landmark complete-case set (out-of-fold predictions)
0.120	0	0.131	0.142	0.153	0.155	day-42 landmark complete-case set (out-of-fold predictions)
0.130	0	0.121	0.134	0.145	0.156	day-42 landmark complete-case set (out-of-fold predictions)
0.140	0	0.111	0.127	0.143	0.152	day-42 landmark complete-case set (out-of-fold predictions)
0.150	0	0.100	0.116	0.140	0.141	day-42 landmark complete-case set (out-of-fold predictions)
0.160	0	0.090	0.128	0.138	0.139	day-42 landmark complete-case set (out-of-fold predictions)
0.170	0	0.079	0.124	0.139	0.140	day-42 landmark complete-case set (out-of-fold predictions)

threshold	treat_none	treat_all	D0: EPS-4 (landmark)	D1: + log2 NLR ratio	D2: + log2 LDH ratio	analysis_set
0.180	0	0.067	0.119	0.136	0.143	day-42 landmark complete-case set (out-of-fold predictions)
0.190	0	0.056	0.115	0.133	0.140	day-42 landmark complete-case set (out-of-fold predictions)
0.200	0	0.044	0.110	0.124	0.130	day-42 landmark complete-case set (out-of-fold predictions)
0.210	0	0.032	0.106	0.121	0.123	day-42 landmark complete-case set (out-of-fold predictions)
0.220	0	0.020	0.101	0.112	0.124	day-42 landmark complete-case set (out-of-fold predictions)
0.230	0	0.007	0.096	0.105	0.114	day-42 landmark complete-case set (out-of-fold predictions)
0.240	0	-0.006	0.091	0.104	0.114	day-42 landmark complete-case set (out-of-fold predictions)
0.250	0	-0.020	0.085	0.099	0.112	day-42 landmark complete-case set (out-of-fold predictions)
0.260	0	-0.033	0.080	0.102	0.108	day-42 landmark complete-case set (out-of-fold predictions)
0.270	0	-0.048	0.075	0.095	0.102	day-42 landmark complete-case set (out-of-fold predictions)
0.280	0	-0.062	0.069	0.093	0.095	day-42 landmark complete-case set (out-of-fold predictions)
0.290	0	-0.077	0.029	0.094	0.093	day-42 landmark complete-case set (out-of-fold predictions)
0.300	0	-0.092	0.034	0.086	0.083	day-42 landmark complete-case set (out-of-fold predictions)
0.310	0	-0.108	0.032	0.077	0.084	day-42 landmark complete-case set (out-of-fold predictions)
0.320	0	-0.125	0.030	0.071	0.083	day-42 landmark complete-case set (out-of-fold predictions)
0.330	0	-0.141	0.028	0.072	0.081	day-42 landmark complete-case set (out-of-fold predictions)
0.340	0	-0.159	0.026	0.080	0.076	day-42 landmark complete-case set (out-of-fold predictions)
0.350	0	-0.176	0.024	0.074	0.073	day-42 landmark complete-case set (out-of-fold predictions)
0.360	0	-0.195	0.022	0.074	0.078	day-42 landmark complete-case set (out-of-fold predictions)
0.370	0	-0.214	0.020	0.063	0.071	day-42 landmark complete-case set (out-of-fold predictions)
0.380	0	-0.233	0.017	0.052	0.065	day-42 landmark complete-case set (out-of-fold predictions)
0.390	0	-0.254	0.015	0.043	0.061	day-42 landmark complete-case set (out-of-fold predictions)
0.400	0	-0.275	0.013	0.042	0.062	day-42 landmark complete-case set (out-of-fold predictions)
0.410	0	-0.296	0.010	0.042	0.062	day-42 landmark complete-case set (out-of-fold predictions)
0.420	0	-0.318	0.008	0.040	0.051	day-42 landmark complete-case set (out-of-fold predictions)
0.430	0	-0.342	0.005	0.037	0.045	day-42 landmark complete-case set (out-of-fold predictions)
0.440	0	-0.366	0.002	0.031	0.046	day-42 landmark complete-case set (out-of-fold predictions)
0.450	0	-0.390	-7.64e-04	0.031	0.043	day-42 landmark complete-case set (out-of-fold predictions)
0.460	0	-0.416	-0.012	0.024	0.041	day-42 landmark complete-case set (out-of-fold predictions)
0.470	0	-0.443	-0.053	0.032	0.033	day-42 landmark complete-case set (out-of-fold predictions)
0.480	0	-0.471	-0.018	0.026	0.022	day-42 landmark complete-case set (out-of-fold predictions)
0.490	0	-0.499	-0.008	0.032	0.019	day-42 landmark complete-case set (out-of-fold predictions)
0.500	0	-0.529	-0.004	0.029	0.013	day-42 landmark complete-case set (out-of-fold predictions)

Table S12a. Sensitivity analyses

Analysis	Restriction	n/events	AUC (95% CI)	Key term	OR (95% CI)	P	Note
EPS-4 for EPD90 (baseline)	Full analysis set (reference)	262/76	0.732 (0.669 to 0.795)	eps4	2.36 (1.75 to 3.17)	<0.001	
EPS-4 for EPD90 (baseline)	Stage IV only	260/75	0.731 (0.667 to 0.795)	eps4	2.34 (1.74 to 3.16)	<0.001	
EPS-4 for EPD90 (baseline)	ICI monotherapy only	252/74	0.730 (0.665 to 0.794)	eps4	2.33 (1.72 to 3.15)	<0.001	
EPS-4 for EPD90 (baseline)	Second or later line, monotherapy (closest to LIPI/EPSILoN derivation)	233/72	0.733 (0.667 to 0.800)	eps4	2.38 (1.74 to 3.25)	<0.001	
EPS-4 for EPD90 (baseline)	Nivolumab monotherapy only	216/65	0.737 (0.669 to 0.806)	eps4	2.42 (1.74 to 3.36)	<0.001	
EPS-4 for EPD90 (baseline)	Chemo-IO patients excluded	252/74	0.730 (0.665 to 0.794)	eps4	2.33 (1.72 to 3.15)	<0.001	
EPS-4 for EPD90 (baseline)	First-line only	27/4	-	-	-	-	not estimable: fewer than 30 patients or 8 events
D2 landmark model (EPD days 43-90)	Full analysis set (reference)	238/56	0.816 (0.751 to 0.881)	eps4	2.28 (1.59 to 3.26)	<0.001	

Analysis	Restriction	n/events	AUC (95% CI)	Key term	OR (95% CI)	P	Note
D2 landmark model (EPD days 43-90)	Stage IV only	236/55	0.812 (0.746 to 0.878)	eps4	2.27 (1.58 to 3.24)	<0.001	
D2 landmark model (EPD days 43-90)	ICI monotherapy only	228/54	0.822 (0.756 to 0.889)	eps4	2.29 (1.58 to 3.31)	<0.001	
D2 landmark model (EPD days 43-90)	Second or later line, monotherapy (closest to LIPI/EPSILoN derivation)	209/52	0.836 (0.769 to 0.902)	eps4	2.21 (1.50 to 3.25)	<0.001	
D2 landmark model (EPD days 43-90)	Nivolumab monotherapy only	194/47	0.849 (0.786 to 0.913)	eps4	2.25 (1.48 to 3.41)	<0.001	
D2 landmark model (EPD days 43-90)	Chemo-IO patients excluded	228/54	0.822 (0.756 to 0.889)	eps4	2.29 (1.58 to 3.31)	<0.001	
D2 landmark model (EPD days 43-90)	First-line only	27/4	-	-	-	-	not estimable: fewer than 30 patients or 8 events
EPS-4 for EPD90	Full analysis set (reference)	262/76	0.732 (0.669 to 0.795)	eps4	2.36 (1.75 to 3.17)	<0.001	
GRIm for EPD90	Full analysis set (reference)	278/81	0.676 (0.611 to 0.741)	grim	2.04 (1.51 to 2.76)	<0.001	
mGPS for EPD90	Full analysis set (reference)	278/81	0.666 (0.603 to 0.730)	mgps	2.64 (1.75 to 3.96)	<0.001	
aLIPI for EPD90	Full analysis set (reference)	278/81	0.645 (0.579 to 0.711)	alipi	2.08 (1.46 to 2.97)	<0.001	
EPSILoN for EPD90	Full analysis set (reference)	252/76	0.610 (0.537 to 0.683)	epsilon	1.53 (1.16 to 2.01)	0.003	
EPS-4 for EPD90	Stage IV only	260/75	0.731 (0.667 to 0.795)	eps4	2.34 (1.74 to 3.16)	<0.001	
GRIm for EPD90	Stage IV only	271/80	0.677 (0.612 to 0.743)	grim	2.04 (1.51 to 2.76)	<0.001	
mGPS for EPD90	Stage IV only	271/80	0.668 (0.604 to 0.732)	mgps	2.63 (1.75 to 3.94)	<0.001	
aLIPI for EPD90	Stage IV only	271/80	0.645 (0.579 to 0.712)	alipi	2.08 (1.45 to 2.97)	<0.001	
EPSILoN for EPD90	Stage IV only	250/75	0.605 (0.532 to 0.679)	epsilon	1.51 (1.14 to 1.99)	0.004	
EPS-4 for EPD90	ICI monotherapy only	252/74	0.730 (0.665 to 0.794)	eps4	2.33 (1.72 to 3.15)	<0.001	
GRIm for EPD90	ICI monotherapy only	263/79	0.680 (0.614 to 0.746)	grim	2.07 (1.52 to 2.81)	<0.001	
mGPS for EPD90	ICI monotherapy only	263/79	0.661 (0.596 to 0.726)	mgps	2.51 (1.67 to 3.78)	<0.001	
aLIPI for EPD90	ICI monotherapy only	263/79	0.654 (0.587 to 0.721)	alipi	2.20 (1.52 to 3.17)	<0.001	
EPSILoN for EPD90	ICI monotherapy only	242/74	0.607 (0.532 to 0.682)	epsilon	1.51 (1.14 to 1.99)	0.004	
EPS-4 for EPD90	Second or later line, monotherapy (closest to LIPI/EPSILoN derivation)	233/72	0.733 (0.667 to 0.800)	eps4	2.38 (1.74 to 3.25)	<0.001	
GRIm for EPD90	Second or later line, monotherapy (closest to LIPI/EPSILoN derivation)	240/75	0.692 (0.625 to 0.760)	grim	2.21 (1.59 to 3.07)	<0.001	
mGPS for EPD90	Second or later line, monotherapy (closest to LIPI/EPSILoN derivation)	240/75	0.662 (0.595 to 0.729)	mgps	2.62 (1.69 to 4.05)	<0.001	
aLIPI for EPD90	Second or later line, monotherapy (closest to LIPI/EPSILoN derivation)	240/75	0.666 (0.598 to 0.735)	alipi	2.35 (1.59 to 3.46)	<0.001	

Analysis	Restriction	n/events	AUC (95% CI)	Key term	OR (95% CI)	P	Note
EPSILoN for EPD90	Second or later line, monotherapy (closest to LIPI/EPSILoN derivation)	223/72	0.619 (0.542 to 0.695)	epsilon	1.57 (1.18 to 2.10)	0.002	
EPS-4 for EPD90	Nivolumab monotherapy only	216/65	0.737 (0.669 to 0.806)	eps4	2.42 (1.74 to 3.36)	<0.001	
GRIm for EPD90	Nivolumab monotherapy only	220/67	0.694 (0.623 to 0.765)	grim	2.18 (1.55 to 3.06)	<0.001	
mGPS for EPD90	Nivolumab monotherapy only	220/67	0.654 (0.583 to 0.724)	mgps	2.47 (1.57 to 3.90)	<0.001	
aLIPI for EPD90	Nivolumab monotherapy only	220/67	0.666 (0.594 to 0.738)	alipi	2.30 (1.54 to 3.44)	<0.001	
EPSILoN for EPD90	Nivolumab monotherapy only	206/65	0.613 (0.531 to 0.695)	epsilon	1.55 (1.15 to 2.08)	0.004	
EPS-4 for EPD90	Chemo-IO patients excluded	252/74	0.730 (0.665 to 0.794)	eps4	2.33 (1.72 to 3.15)	<0.001	
GRIm for EPD90	Chemo-IO patients excluded	263/79	0.680 (0.614 to 0.746)	grim	2.07 (1.52 to 2.81)	<0.001	
mGPS for EPD90	Chemo-IO patients excluded	263/79	0.661 (0.596 to 0.726)	mgps	2.51 (1.67 to 3.78)	<0.001	
aLIPI for EPD90	Chemo-IO patients excluded	263/79	0.654 (0.587 to 0.721)	alipi	2.20 (1.52 to 3.17)	<0.001	
EPSILoN for EPD90	Chemo-IO patients excluded	242/74	0.607 (0.532 to 0.682)	epsilon	1.51 (1.14 to 1.99)	0.004	
EPS-4 for EPD90	First-line only	27/4	-	-	-	-	not estimable: fewer than 30 patients or 8 events
GRIm for EPD90	First-line only	36/6	-	-	-	-	not estimable: fewer than 30 patients or 8 events
mGPS for EPD90	First-line only	36/6	-	-	-	-	not estimable: fewer than 30 patients or 8 events
aLIPI for EPD90	First-line only	36/6	-	-	-	-	not estimable: fewer than 30 patients or 8 events
EPSILoN for EPD90	First-line only	27/4	—	epsilon	—	—	not estimable: fewer than 30 patients or 8 events
EPS-4	Primary endpoint: EPD90	262/76	0.732 (0.669 to 0.795)	eps4	2.36 (1.75 to 3.17)	<0.001	
EPS-4	EPD60 (baseline models)	262/37	0.721 (0.638 to 0.804)	eps4	2.25 (1.56 to 3.24)	<0.001	
EPS-4	EPD120 (baseline models)	261/113	0.741 (0.682 to 0.800)	eps4	2.52 (1.89 to 3.36)	<0.001	
EPS-4	Progression only within 90 days (deaths censored)	248/60	0.719 (0.648 to 0.791)	eps4	2.22 (1.62 to 3.04)	<0.001	
EPS-4	All-cause death within 90 days (separate outcome)	262/44	0.756 (0.689 to 0.824)	eps4	2.59 (1.80 to 3.72)	<0.001	
D2	Primary landmark endpoint: EPD days 43-90	238/56	0.816 (0.751 to 0.881)	eps4	2.28 (1.59 to 3.26)	<0.001	
D2	EPD days 43-60 ONLY (a shorter residual-risk window, not all 60-day events)	238/17	0.817 (0.730 to 0.904)	eps4	2.28 (1.59 to 3.26)	<0.001	
D2 with a 45-day grace landmark	SENSITIVITY: landmark moved from day 42 to day 45	235/53	0.811 (0.743 to 0.879)	eps4	2.22 (1.55 to 3.17)	<0.001	

Analysis	Restriction	n/events	AUC (95% CI)	Key term	OR (95% CI)	P	Note
EPSILoN	Primary corrected definition (smoking <40 pack-years)	252/76	0.610 (0.537 to 0.683)	epsilon	1.53 (1.16 to 2.01)	0.003	ECOG >=2, liver metastasis, LDH >=400 U/L, NLR >=4
EPSILoN	Previous implementation (smoking <43 pack-years)	252/76	0.594 (0.520 to 0.668)	epsilon	1.43 (1.08 to 1.88)	0.011	Sensitivity analysis only; all other components unchanged

Table S12b. Continuous versus prespecified categorical dynamic model

model	n	events	prevalence	auc	auc_lo	auc_hi	pr_auc	pr_auc_lo	pr_auc_hi	brier	brier_scaled	calib_intercept	calib_slope
D2 continuous (log2 ratios)	238	56	0.235294117647059	0.816326530612245	0.751161476058784	0.881491585165706	0.62596112094673	0.499463708556573	0.737231357527651	0.13397692685276	0.255397464222163	5.59331072856133e-11	1.00000000000016
D2-categorical (prespecified decrease indicators)	238	56	0.235294117647059	0.787382260596546	0.71930867213479	0.855455849058302	0.546227364489003	0.407149350906935	0.669103009153537	0.143108070617076	0.204649376762788	2.24585515772465e-11	1.00000000000001

Table S12c. Firth penalised adjusted landmark model

term	beta	or	lo	hi	p	or_ci
(Intercept)	-3.99905312756539	0.0183329896755147	0.00368857131662209	0.0911188863091636	1.01731005146566e-06	0.02 (0.00 to 0.09)
eps4	0.84003048671864	2.31643759628585	1.60063138956538	3.352354060071	8.41604208805573e-06	2.32 (1.60 to 3.35)
log2_nlr_ratio	0.781312895772877	2.18433819213404	1.49719987426288	3.18683792300243	5.02857036430583e-05	2.18 (1.50 to 3.19)
log2_ldh_ratio	0.818553107828332	2.26721704458812	1.11777614721791	4.59866059949908	0.0232941251947559	2.27 (1.12 to 4.60)
ecog_ge2	-0.0862808756721586	0.917336537515329	0.376624239456975	2.2343392562144	0.849337742333844	0.92 (0.38 to 2.23)
histology3Squamous	0.817244934124156	2.26425306998824	1.0589611410212	4.84138819296727	0.0350524345904645	2.26 (1.06 to 4.84)
histology3NSCLC-NOS	-0.674718369608498	0.50929983472356	0.0836074664139131	3.10243011509653	0.464238083210697	0.51 (0.08 to 3.10)
regimen_mono_vs_chemoioChemo-IO	1.93215219932636	6.90435380830973	0.960235415948764	49.6441817480981	0.0548976973647558	6.90 (0.96 to 49.64)
line_first_vs_laterSecond or later	0.883466191881366	2.41927084701493	0.654472105313617	8.94288906081287	0.185349136809	2.42 (0.65 to 8.94)

Note. Separation sensitivity analysis.

Table S12d. Exploratory Youden cut-points

variable	cutpoint	sensitivity	specificity	auc	warning
log2 NLR ratio	0.660235289930788	0.553571428571429	0.851648351648352	0.730278649921507	EXPLORATORY: cut-point optimised in the same data; not validated, not for clinical use
log2 LDH ratio	-0.00606813359844287	0.714285714285714	0.554945054945055	0.675824175824176	EXPLORATORY: cut-point optimised in the same data; not validated, not for clinical use
NLR ratio	1.58036676851231	0.553571428571429	0.851648351648352	0.730278649921507	EXPLORATORY: cut-point optimised in the same data; not validated, not for clinical use
LDH ratio	0.995804925743882	0.714285714285714	0.554945054945055	0.675824175824176	EXPLORATORY: cut-point optimised in the same data; not validated, not for clinical use

Note. EXPLORATORY ONLY. These thresholds were optimised in the same data, are optimistically biased, and are not for clinical use.

Table S12e. Exact-date versus stored endpoint definition

analysis	restriction	n	events	auc	auc_lo	auc_hi	key_term	or	or_lo	or_hi	p	note
EPS-4	EPD90 derived from exact dates (primary)	262	76	0.732243916242218	0.669048187826123	0.795439644658314	eps4	2.35697515721594	1.74972450802341	3.17497518395553	1.69421217241872e-08	
EPS-4	EPD90 taken from the workbook’s stored EP/non-EP column	262	76	0.732243916242218	0.669048187826123	0.795439644658314	eps4	2.35697515721594	1.74972450802341	3.17497518395553	1.69421217241872e-08	

Table S12f. PD-L1 adjusted sensitivity model

term	or	lo	hi	p	or_ci
(Intercept)	0.101634410477565	0.0470572744966531	0.219510235210433	5.8941833619612e-09	0.10 (0.05 to 0.22)
eps4	2.42582044813837	1.72349735733213	3.41433934991073	3.7467471983212e-07	2.43 (1.72 to 3.41)
pdl1_cat1-49%	0.935870713160105	0.407472930594764	2.14947773456355	0.875852610014204	0.94 (0.41 to 2.15)
pdl1_cat>=50%	0.452026187368776	0.188359637690911	1.08477419351615	0.0754354528620468	0.45 (0.19 to 1.08)

Table S12g. Multiple-imputation model

term	or	lo	hi	p	m	or_ci	p_fmt	note
(Intercept)	0.0347094644906131	0.00924527891351033	0.130309419163613	1.02708770395562e-06	30	0.03 (0.01 to 0.13)	<0.001	SUPPORTING analysis; the primary score benchmarking is complete-case. EPS-4 is recomputed from imputed components inside every imputed data set.
eps4_mi	2.51320225763487	1.85004549705822	3.41407040952477	9.85674699265283e-09	30	2.51 (1.85 to 3.41)	<0.001	SUPPORTING analysis; the primary score benchmarking is complete-case. EPS-4 is recomputed from imputed components inside every imputed data set.
ecog_ge2	0.953861345665425	0.460781175813587	1.97458471507253	0.898391235922679	30	0.95 (0.46 to 1.97)	0.898	SUPPORTING analysis; the primary score benchmarking is complete-case. EPS-4 is recomputed from imputed components inside every imputed data set.
histology3Squamous	1.17674261654957	0.644229825109589	2.14942421420549	0.595267322964383	30	1.18 (0.64 to 2.15)	0.595	SUPPORTING analysis; the primary score benchmarking is complete-case. EPS-4 is recomputed from imputed components inside every imputed data set.
histology3NSCLC-NOS	0.716080044324137	0.225587528892465	2.27304511200923	0.569689357192358	30	0.72 (0.23 to 2.27)	0.570	SUPPORTING analysis; the primary score benchmarking is complete-case. EPS-4 is recomputed from imputed components inside every imputed data set.
regimen_mono_vs_chemoioChemo-IO	1.39094857079061	0.272759332598704	7.09320523756714	0.690366774105159	30	1.39 (0.27 to 7.09)	0.690	SUPPORTING analysis; the primary score benchmarking is complete-case. EPS-4 is recomputed from imputed components inside every imputed data set.
line_first_vs_laterSecond or later	2.71097274317429	0.897109271792103	8.19228319818	0.0769445420116836	30	2.71 (0.90 to 8.19)	0.077	SUPPORTING analysis; the primary score benchmarking is complete-case. EPS-4 is recomputed from imputed components inside every imputed data set.

Table S12h. Complete-case versus multiple-imputation estimates

term	or_complete_case	lo_cc	hi_cc	n_complete_case	or_mi	lo_mi	hi_mi	cc_ci	mi_ci
(Intercept)	0.0219923907425067	0.00478151475817142	0.101153143937186	259	0.0347094644906131	0.00924527891351033	0.130309419163613	0.02 (0.00 to 0.10)	0.03 (0.01 to 0.13)
eps4	2.45325840323872	1.80006036403951	3.34348609263045	259	2.51320225763487	1.85004549705822	3.41407040952477	2.45 (1.80 to 3.34)	2.51 (1.85 to 3.41)
ecog_ge2	0.950825436340389	0.451511062418845	2.00231862658822	259	0.953861345665425	0.460781175813587	1.97458471507253	0.95 (0.45 to 2.00)	0.95 (0.46 to 1.97)
histology3Squamous	1.32927106461446	0.705777863489849	2.50356614258797	259	1.17674261654957	0.644229825109589	2.14942421420549	1.33 (0.71 to 2.50)	1.18 (0.64 to 2.15)
histology3NSCLC-NOS	0.815351879149478	0.254136033576418	2.61591667059949	259	0.716080044324137	0.225587528892465	2.27304511200923	0.82 (0.25 to 2.62)	0.72 (0.23 to 2.27)
regimen_mono_vs_chemoioChemo-IO	2.22447983639661	0.31508719478804	15.7045751918411	259	1.39094857079061	0.272759332598704	7.09320523756714	2.22 (0.32 to 15.70)	1.39 (0.27 to 7.09)
line_first_vs_laterSecond or later	4.16050874706462	1.11429547286159	15.5343294987534	259	2.71097274317429	0.897109271792103	8.19228319818	4.16 (1.11 to 15.53)	2.71 (0.90 to 8.19)

Table S12i. Inverse-probability-weighted sensitivity analysis

term	or_ipw	lo	hi	p	n	events	weight_min	weight_max	or_ci	note
(Intercept)	0.058489231536102	0.0275072570737836	0.124366824235056	1.63331294494425e-13	235	56	1.00000000542581	1.20476998773785	0.06 (0.03 to 0.12)	Inverse-probability weighting for day-42 laboratory availability; standard errors are not robust-corrected, so the CIs are approximate
eps4	2.25636705366422	1.58847075792083	3.20509033954474	5.51300031561588e-06	235	56	1.00000000542581	1.20476998773785	2.26 (1.59 to 3.21)	Inverse-probability weighting for day-42 laboratory availability; standard errors are not robust-corrected, so the CIs are approximate
log2_nlr_ratio	2.11428438938817	1.48333952422685	3.0136043745888	3.46522375980565e-05	235	56	1.00000000542581	1.20476998773785	2.11 (1.48 to 3.01)	Inverse-probability weighting for day-42 laboratory availability; standard errors are not robust-corrected, so the CIs are approximate
log2_ldh_ratio	2.09609797299187	1.08067735455065	4.06562300383124	0.0285571003593947	235	56	1.00000000542581	1.20476998773785	2.10 (1.08 to 4.07)	Inverse-probability weighting for day-42 laboratory availability; standard errors are not robust-corrected, so the CIs are approximate

Table S13. Subgroup interaction models

Analysis	Modifier	Stratum	n/events	OR (95% CI)	Interaction P
EPS-4 for EPD90	Regimen (mono vs chemo-IO)	ICI monotherapy	266/80	2.33 (1.72 to 3.15)	0.728
EPS-4 for EPD90	Regimen (mono vs chemo-IO)	Chemo-IO	16/3	-	0.728
EPS-4 for EPD90	Treatment line	First line	37/7	2.92 (0.80 to 10.60)	0.761
EPS-4 for EPD90	Treatment line	Second or later	245/76	2.39 (1.75 to 3.26)	0.761

Analysis	Modifier	Stratum	n/events	OR (95% CI)	Interaction P
EPS-4 for EPD90	Histology	Adenocarcinoma	161/47	2.11 (1.44 to 3.09)	0.547
EPS-4 for EPD90	Histology	Squamous	100/30	2.65 (1.54 to 4.54)	0.547
EPS-4 for EPD90	Histology	NSCLC-NOS	21/6	-	0.547
EPS-4 for EPD90	PD-L1 category	<1%	118/39	2.53 (1.62 to 3.95)	0.520
EPS-4 for EPD90	PD-L1 category	1-49%	49/12	3.25 (1.34 to 7.84)	0.520
EPS-4 for EPD90	PD-L1 category	>=50%	53/13	1.75 (0.88 to 3.48)	0.520
EPS-4 for EPD90	ECOG >= 2	0	231/67	2.45 (1.76 to 3.41)	0.469
EPS-4 for EPD90	ECOG >= 2	1	48/16	1.83 (0.90 to 3.70)	0.469
log2 NLR ratio for EPD days 43-90	Regimen (mono vs chemo-IO)	ICI monotherapy	228/54	2.54 (1.77 to 3.64)	0.053
log2 NLR ratio for EPD days 43-90	Regimen (mono vs chemo-IO)	Chemo-IO	10/2	-	0.053
log2 NLR ratio for EPD days 43-90	Treatment line	First line	27/4	-	<0.001
log2 NLR ratio for EPD days 43-90	Treatment line	Second or later	211/52	3.09 (2.05 to 4.66)	<0.001
log2 NLR ratio for EPD days 43-90	Histology	Adenocarcinoma	135/29	2.33 (1.50 to 3.61)	0.554
log2 NLR ratio for EPD days 43-90	Histology	Squamous	86/25	2.54 (1.40 to 4.60)	0.554
log2 NLR ratio for EPD days 43-90	Histology	NSCLC-NOS	17/2	-	0.554

Note. Exploratory. Only the interaction test is interpretable; stratum-specific estimates have wide confidence intervals.

Table S14a. Cox models

Model	Time origin	Term	n/events	HR (95% CI)	P	C-index	Global PH P
PFS: EPS-4 (per point)	ICI start	eps4	262/216	1.48 (1.30 to 1.67)	<0.001	0.6431	<0.001
PFS: GRIm (per point)	ICI start	grim	278/229	1.41 (1.22 to 1.62)	<0.001	0.5977	0.013
PFS: mGPS (per point)	ICI start	mgps	278/229	1.47 (1.22 to 1.78)	<0.001	0.593	<0.001
PFS: aLIPI (per point)	ICI start	alipi	278/229	1.50 (1.26 to 1.79)	<0.001	0.588	0.108
PFS: EPS-4 >= 2 vs 0-1	ICI start	eps4_ge2	262/216	2.08 (1.58 to 2.72)	<0.001	0.6163	<0.001
OS: EPS-4 (per point)	ICI start	eps4	262/191	1.46 (1.28 to 1.67)	<0.001	0.6452	<0.001
OS: GRIm (per point)	ICI start	grim	278/199	1.42 (1.22 to 1.65)	<0.001	0.6069	0.003
OS: mGPS (per point)	ICI start	mgps	278/199	1.52 (1.24 to 1.87)	<0.001	0.5998	<0.001
OS: aLIPI (per point)	ICI start	alipi	278/199	1.52 (1.26 to 1.84)	<0.001	0.5972	0.026
OS: EPS-4 >= 2 vs 0-1	ICI start	eps4_ge2	262/191	1.91 (1.43 to 2.55)	<0.001	0.6116	<0.001
PFS: EPS-4 (per point)	day 42 (delayed entry)	eps4	238/193	1.40 (1.23 to 1.61)	<0.001	0.6281	0.004
PFS: log2 NLR ratio (per doubling)	day 42 (delayed entry)	log2_nlr_ratio	238/193	1.64 (1.41 to 1.92)	<0.001	0.6535	<0.001
PFS: log2 LDH ratio (per doubling)	day 42 (delayed entry)	log2_ldh_ratio	238/193	2.28 (1.74 to 2.99)	<0.001	0.6323	0.284
PFS: NLR decreased (ratio < 1)	day 42 (delayed entry)	nlr_decrease	238/193	0.56 (0.42 to 0.74)	<0.001	0.6008	<0.001
PFS: Dual adverse (NLR +50% AND LDH +25%)	day 42 (delayed entry)	dual_adverse	238/193	4.62 (2.98 to 7.16)	<0.001	0.5675	0.153

Model	Time origin	Term	n/events	HR (95% CI)	P	C-index	Global PH P
PFS: D2 terms jointly	day 42 (delayed entry)	eps4	238/193	1.60 (1.39 to 1.85)	<0.001	0.7159	0.010
PFS: D2 terms jointly	day 42 (delayed entry)	log2_nlr_ratio	238/193	1.62 (1.39 to 1.90)	<0.001	0.7159	0.010
PFS: D2 terms jointly	day 42 (delayed entry)	log2_ldh_ratio	238/193	1.99 (1.55 to 2.55)	<0.001	0.7159	0.010
OS: EPS-4 (per point)	day 42 (delayed entry)	eps4	238/168	1.38 (1.20 to 1.59)	<0.001	0.6269	<0.001
OS: log2 NLR ratio (per doubling)	day 42 (delayed entry)	log2_nlr_ratio	238/168	1.72 (1.46 to 2.03)	<0.001	0.6729	<0.001
OS: log2 LDH ratio (per doubling)	day 42 (delayed entry)	log2_ldh_ratio	238/168	2.36 (1.77 to 3.13)	<0.001	0.6421	0.040
OS: NLR decreased (ratio < 1)	day 42 (delayed entry)	nlr_decrease	238/168	0.53 (0.39 to 0.72)	<0.001	0.6092	<0.001
OS: Dual adverse (NLR +50% AND LDH +25%)	day 42 (delayed entry)	dual_adverse	238/168	5.81 (3.70 to 9.14)	<0.001	0.5799	0.067
OS: D2 terms jointly	day 42 (delayed entry)	eps4	238/168	1.61 (1.38 to 1.87)	<0.001	0.7313	<0.001
OS: D2 terms jointly	day 42 (delayed entry)	log2_nlr_ratio	238/168	1.74 (1.47 to 2.05)	<0.001	0.7313	<0.001
OS: D2 terms jointly	day 42 (delayed entry)	log2_ldh_ratio	238/168	2.00 (1.53 to 2.61)	<0.001	0.7313	<0.001

Note. Models containing day-42 information use delayed entry at day 42. Comparator scores were benchmarked against EPD90 only; the survival analyses focus on EPS-4 and its dynamic updates. Proportional-hazards diagnostics are detailed in Table S14e; where the assumption was violated, hazard ratios should be read as time-averaged associations rather than constant effects.

Table S14b. Kaplan-Meier summaries

Endpoint	Time origin	Group	n/events	Median months (95% CI)	6-month	12-month	Log-rank P
PFS	ICI start	EPS-4 0-1	129/97	9.2 (6.9 to 11.5)	68.2 (60.6 to 76.7)	39.1 (31.4 to 48.5)	<0.001
PFS	ICI start	EPS-4 2	85/74	3.5 (3.1 to 5.6)	35.3 (26.5 to 47.1)	25.9 (18.1 to 37.1)	<0.001
PFS	ICI start	EPS-4 3-4	48/45	2.7 (2.2 to 3.3)	19.9 (11.2 to 35.5)	13.3 (6.3 to 27.8)	<0.001
OS	ICI start	EPS-4 0-1	129/86	20.9 (14.9 to 26.1)	85.3 (79.4 to 91.6)	63.9 (56.0 to 72.8)	<0.001
OS	ICI start	EPS-4 2	85/64	8.1 (5.7 to 13.6)	56.8 (47.1 to 68.6)	45.2 (35.6 to 57.5)	<0.001
OS	ICI start	EPS-4 3-4	48/41	3.8 (3.1 to 6.7)	36.3 (24.8 to 53.2)	27.3 (17.0 to 43.8)	<0.001
PFS	ICI start	Low risk (0-1)	231/185	6.2 (5.3 to 8.0)	52.4 (46.3 to 59.2)	33.1 (27.6 to 39.8)	<0.001
PFS	ICI start	High risk (2-3)	47/44	2.9 (2.2 to 6.6)	33.5 (22.3 to 50.3)	20.1 (11.3 to 35.9)	<0.001
OS	ICI start	Low risk (0-1)	231/159	14.4 (11.6 to 20.3)	71.2 (65.6 to 77.3)	54.4 (48.2 to 61.3)	<0.001
OS	ICI start	High risk (2-3)	47/40	5.2 (3.3 to 13.4)	48.4 (36.0 to 65.2)	37.4 (25.7 to 54.4)	<0.001
PFS	ICI start	Good	149/114	7.4 (6.2 to 10.2)	59.1 (51.7 to 67.5)	36.7 (29.7 to 45.3)	<0.001
PFS	ICI start	Intermediate	89/77	4.6 (3.1 to 6.6)	41.6 (32.5 to 53.2)	25.7 (18.0 to 36.6)	<0.001
PFS	ICI start	Poor	40/38	3.1 (2.4 to 5.0)	29.0 (17.7 to 47.5)	21.1 (11.5 to 38.9)	<0.001
OS	ICI start	Good	149/96	17.9 (12.9 to 25.1)	79.7 (73.5 to 86.5)	59.9 (52.4 to 68.5)	<0.001
OS	ICI start	Intermediate	89/69	8.1 (5.8 to 15.0)	56.1 (46.6 to 67.4)	43.4 (34.2 to 55.1)	<0.001
OS	ICI start	Poor	40/34	5.0 (3.3 to 14.1)	46.3 (33.0 to 65.1)	38.1 (25.4 to 57.2)	<0.001
PFS	ICI start	Favourable (0)	38/29	8.6 (6.2 to 23.5)	65.8 (52.3 to 82.7)	38.0 (25.1 to 57.5)	0.036
PFS	ICI start	Intermediate (1-2)	178/149	4.8 (3.8 to 6.8)	46.6 (39.8 to 54.6)	28.7 (22.7 to 36.1)	0.036
PFS	ICI start	Poor (3-5)	36/32	3.0 (2.2 to 5.7)	29.5 (17.7 to 49.3)	23.6 (13.0 to 43.0)	0.036
OS	ICI start	Favourable (0)	38/23	22.5 (12.1 to -)	73.7 (60.9 to 89.1)	65.2 (51.5 to 82.5)	0.004
OS	ICI start	Intermediate (1-2)	178/132	12.3 (9.5 to 15.7)	68.8 (62.3 to 76.0)	50.6 (43.7 to 58.6)	0.004
OS	ICI start	Poor (3-5)	36/31	5.0 (2.9 to 11.9)	43.6 (29.9 to 63.5)	29.0 (17.3 to 48.8)	0.004
PFS from day 42	day 42 (clock reset)	Low tertile	80/53	12.4 (8.7 to 22.8)	64.8 (55.1 to 76.2)	51.9 (41.9 to 64.2)	<0.001
PFS from day 42	day 42 (clock reset)	Middle tertile	79/63	7.4 (4.8 to 10.4)	54.4 (44.5 to 66.6)	34.2 (25.2 to 46.4)	<0.001

Endpoint	Time origin	Group	n/events	Median months (95% CI)	6-month	12-month	Log-rank P
PFS from day 42	day 42 (clock reset)	High tertile	79/77	1.6 (1.4 to 1.9)	11.8 (6.4 to 21.7)	6.5 (2.8 to 15.3)	<0.001
OS from day 42	day 42 (clock reset)	Low tertile	80/42	28.1 (23.4 to -)	85.9 (78.5 to 94.0)	74.1 (64.9 to 84.5)	<0.001
OS from day 42	day 42 (clock reset)	Middle tertile	79/58	16.0 (12.0 to 23.7)	86.0 (78.6 to 94.0)	58.9 (48.9 to 70.9)	<0.001
OS from day 42	day 42 (clock reset)	High tertile	79/68	2.9 (2.2 to 4.6)	30.6 (21.7 to 43.1)	21.8 (14.1 to 33.8)	<0.001
PFS from day 42	day 42 (clock reset)	NLR decreased	111/83	9.1 (6.7 to 13.4)	60.0 (51.5 to 69.9)	42.2 (33.8 to 52.6)	<0.001
PFS from day 42	day 42 (clock reset)	NLR stable or increased	127/110	2.3 (1.8 to 3.4)	29.9 (22.9 to 39.0)	21.3 (15.2 to 29.7)	<0.001
OS from day 42	day 42 (clock reset)	NLR decreased	111/69	22.2 (14.7 to 27.2)	85.1 (78.6 to 92.1)	66.8 (58.4 to 76.4)	<0.001
OS from day 42	day 42 (clock reset)	NLR stable or increased	127/99	7.1 (4.5 to 11.5)	53.1 (45.0 to 62.6)	39.1 (31.4 to 48.8)	<0.001

Table S14c. Overall cohort median PFS and OS

endpoint	n	events	median_months	lo	hi	median_ci
PFS (whole baseline cohort)	282	232	5.71663244353183	4.56673511293635	6.86652977412731	5.7 (4.6 to 6.9)
OS (whole baseline cohort)	282	200	12.3860369609856	10.1190965092402	15.7043121149897	12.4 (10.1 to 15.7)

Table S14d. Restricted mean survival time differences

endpoint	group	reference	tau_months	rmst_group	rmst_reference	rmst_difference	note
PFS by EPS-4 group (from ICI start)	EPS-4 2	EPS-4 0-1	6	3.925	5.132	-1.207	RMST difference in months; reported because proportional hazards cannot be assumed throughout
PFS by EPS-4 group (from ICI start)	EPS-4 3-4	EPS-4 0-1	6	3.036	5.132	-2.096	RMST difference in months; reported because proportional hazards cannot be assumed throughout
PFS by EPS-4 group (from ICI start)	EPS-4 2	EPS-4 0-1	12	5.667	8.191	-2.524	RMST difference in months; reported because proportional hazards cannot be assumed throughout
PFS by EPS-4 group (from ICI start)	EPS-4 3-4	EPS-4 0-1	12	4.054	8.191	-4.137	RMST difference in months; reported because proportional hazards cannot be assumed throughout
OS by EPS-4 group (from ICI start)	EPS-4 2	EPS-4 0-1	6	4.766	5.625	-0.859	RMST difference in months; reported because proportional hazards cannot be assumed throughout
OS by EPS-4 group (from ICI start)	EPS-4 3-4	EPS-4 0-1	6	3.863	5.625	-1.762	RMST difference in months; reported because proportional hazards cannot be assumed throughout
OS by EPS-4 group (from ICI start)	EPS-4 2	EPS-4 0-1	12	7.729	10.079	-2.350	RMST difference in months; reported because proportional hazards cannot be assumed throughout
OS by EPS-4 group (from ICI start)	EPS-4 3-4	EPS-4 0-1	12	5.691	10.079	-4.388	RMST difference in months; reported because proportional hazards cannot be assumed throughout
PFS by D2 risk tertile (from day 42)	Middle tertile	Low tertile	6	4.479	5.049	-0.571	RMST difference in months; reported because proportional hazards cannot be assumed throughout
PFS by D2 risk tertile (from day 42)	High tertile	Low tertile	6	2.137	5.049	-2.912	RMST difference in months; reported because proportional hazards cannot be assumed throughout
PFS by D2 risk tertile (from day 42)	Middle tertile	Low tertile	12	7.060	8.575	-1.515	RMST difference in months; reported because proportional hazards cannot be assumed throughout
PFS by D2 risk tertile (from day 42)	High tertile	Low tertile	12	2.644	8.575	-5.931	RMST difference in months; reported because proportional hazards cannot be assumed throughout
OS by D2 risk tertile (from day 42)	Middle tertile	Low tertile	6	5.545	5.765	-0.221	RMST difference in months; reported because proportional hazards cannot be assumed throughout
OS by D2 risk tertile (from day 42)	High tertile	Low tertile	6	3.395	5.765	-2.370	RMST difference in months; reported because proportional hazards cannot be assumed throughout
OS by D2 risk tertile (from day 42)	Middle tertile	Low tertile	12	9.819	10.559	-0.739	RMST difference in months; reported because proportional hazards cannot be assumed throughout
OS by D2 risk tertile (from day 42)	High tertile	Low tertile	12	4.945	10.559	-5.614	RMST difference in months; reported because proportional hazards cannot be assumed throughout

Table S14e. Schoenfeld residual tests

model	time_origin	term	chisq	df	p	p_fmt
PFS ~ EPS-4	ICI start	eps4	11.979	1	5.38e-04	<0.001
PFS ~ EPS-4	ICI start	GLOBAL	11.979	1	5.38e-04	<0.001
OS ~ EPS-4	ICI start	eps4	15.948	1	6.51e-05	<0.001
OS ~ EPS-4	ICI start	GLOBAL	15.948	1	6.51e-05	<0.001
PFS ~ D2 terms	day 42 (delayed entry)	eps4	1.871	1	0.171	0.171
PFS ~ D2 terms	day 42 (delayed entry)	log2_nlr_ratio	7.605	1	0.006	0.006
PFS ~ D2 terms	day 42 (delayed entry)	log2_ldh_ratio	0.358	1	0.549	0.549
PFS ~ D2 terms	day 42 (delayed entry)	GLOBAL	11.329	3	0.010	0.010
OS ~ D2 terms	day 42 (delayed entry)	eps4	4.729	1	0.030	0.030
OS ~ D2 terms	day 42 (delayed entry)	log2_nlr_ratio	14.145	1	1.69e-04	<0.001
OS ~ D2 terms	day 42 (delayed entry)	log2_ldh_ratio	1.372	1	0.241	0.241
OS ~ D2 terms	day 42 (delayed entry)	GLOBAL	23.603	3	3.02e-05	<0.001

Table S15a. Observations flagged by prespecified influence diagnostics

model	anonymised_row	cooks_d	leverage	max_abs_dfbeta	flagged
D2 landmark model	73	0.022	0.085	0.115	TRUE
D2 landmark model	97	0.030	0.017	0.043	TRUE
D2 landmark model	98	0.035	0.038	0.092	TRUE
D2 landmark model	100	0.018	0.007	0.061	TRUE
D2 landmark model	106	0.026	0.036	0.079	TRUE
D2 landmark model	149	0.033	0.009	0.086	TRUE
D2 landmark model	150	0.020	0.044	0.080	TRUE
D2 landmark model	158	0.052	0.024	0.048	TRUE
D2 landmark model	159	0.032	0.044	0.060	TRUE
D2 landmark model	171	0.052	0.005	0.074	TRUE
D2 landmark model	174	0.034	0.068	0.116	TRUE
D2 landmark model	198	0.035	0.008	0.084	TRUE
D2 landmark model	204	0.022	0.055	0.099	TRUE
D2 landmark model	223	0.025	0.070	0.060	TRUE
D2 landmark model	233	0.029	0.111	0.080	TRUE
D2 landmark model	235	0.021	0.010	0.035	TRUE
B1 baseline model	8	0.016	0.010	0.089	TRUE
B1 baseline model	30	0.024	0.066	0.233	TRUE
B1 baseline model	79	0.017	0.024	0.088	TRUE
B1 baseline model	157	0.024	0.066	0.233	TRUE

Note. The displayed rows are a subset of flagged observations. Table S15b reports exclusion of 16 observations for D2 and 17 for B1. Row identifiers are anonymised positions, not patient identifiers. No observation was deleted from a primary analysis; the sensitivity refit does not establish that coefficient magnitudes are unaffected by influential observations.

Table S15b. Sensitivity refit with influential points removed

model	term	or_all	or_influential_removed	n_all	n_removed	note
D2 landmark model	(Intercept)	0.0561076304926881	0.0122015425776551	238	16	stability check only; the primary estimates are those from the full data
D2 landmark model	eps4	2.28015461341278	4.29453469186334	238	16	stability check only; the primary estimates are those from the full data
D2 landmark model	log2_nlr_ratio	2.14038146273457	2.83244932611842	238	16	stability check only; the primary estimates are those from the full data
D2 landmark model	log2_ldh_ratio	2.09296088342862	4.63122511959257	238	16	stability check only; the primary estimates are those from the full data
B1 baseline model	(Intercept)	0.0219923907425068	1.21834824131635e-09	259	17	stability check only; the primary estimates are those from the full data
B1 baseline model	ecog_ge2	0.95082543634039	0.853691624932576	259	17	stability check only; the primary estimates are those from the full data
B1 baseline model	histology3Squamous	1.32927106461446	1.52445863245877	259	17	stability check only; the primary estimates are those from the full data
B1 baseline model	histology3NSCLC-NOS	0.815351879149477	0.3593087757357	259	17	stability check only; the primary estimates are those from the full data
B1 baseline model	regimen_mono_vs_chemoioChemo-IO	2.22447983639659	1.89045804287675e-06	259	17	stability check only; the primary estimates are those from the full data
B1 baseline model	line_first_vs_laterSecond or later	4.16050874706462	34688696.093411	259	17	stability check only; the primary estimates are those from the full data
B1 baseline model	eps4	2.45325840323872	3.39324817303513	259	17	stability check only; the primary estimates are those from the full data

Table S16. Reconciliation with the previous report's 256/61 landmark cohort

step	n
EPD90-classifiable patients	282
... with week-6 laboratories available	269
LEGACY landmark: stored PFS months x 30.4375 > 42	256
LEGACY events during days 43-90	61
EXACT-DATE landmark: first-event day > 42 (same week-6 subset)	253
EXACT-DATE events during days 43-90 (same subset)	60
Patients excluded pre-day-42 by exact dates but NOT by stored months	3

step	n
Patients excluded pre-day-42 by stored months but NOT by exact dates	0
LEGACY landmark applied to the whole EPD90 cohort (no week-6 filter)	265
EXACT-DATE landmark applied to the whole EPD90 cohort	262

Figure S1. Calendar-year accrual in the EPD90-classifiable cohort

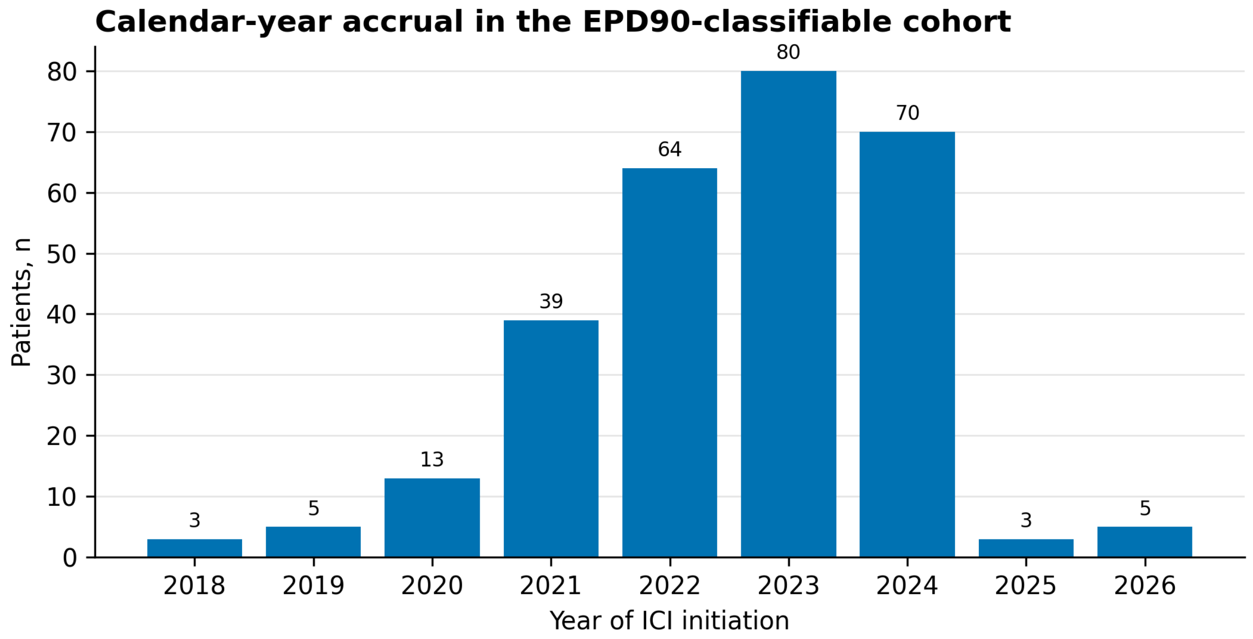

Figure S1. Number of EPD90-classifiable patients by calendar year of immune-checkpoint inhibitor initiation. The small counts in 2025–2026 reflect the source cohort and should be considered when judging temporal transportability; no temporal validation claim is made.
